# Intestinal metabolite TMAO promotes CKD progression by stimulating macrophage M2 polarization through histone H4 lysine 12 lactylation

**DOI:** 10.1038/s41418-025-01554-z

**Published:** 2025-08-19

**Authors:** Youzhou Tang, Yuxin Li, Xinyu Yang, Tianze Lu, Xinran Wang, Zhi Li, Jun Liu, Jianwen Wang

**Affiliations:** 1 Department of Nephrology, The Third Xiangya Hospital, Central South University, Changsha, China; 2 The Critical Kidney Disease Research Center of Central South University, Changsha, China

**Keywords:** Epigenetics, Kidney diseases

## Abstract

Chronic kidney disease (CKD) progression is tightly associated with renal fibrosis, which is regulated by macrophage M2 polarization. The intestinal metabolite trimethylamine N-oxide (TMAO) has been reported to promote CKD, yet its underlying mechanism remains unclear. Here, we elucidated a mechanism wherein TMAO excreted through the kidneys alters the pyruvate metabolism of renal tubular epithelial cells, resulting in the production of lactic acid. Local lactic acid accumulation in the kidney promotes adjacent macrophage M2 polarization, a process speculated to be mediated by specific lactylation of macrophage genes. Through lactylation omics analysis, we identified histone H4 lysine 12 (H4K12) as the most significantly up-regulated lysine residue subjected to lactylation. Subsequent chromatin immunoprecipitation sequencing (ChIP-seq) assays revealed H4K12 lactylation on several glycometabolism gene promoters and genes. Furthermore, we found that this lactylation-mediated epigenetic regulation requires the assistance of the “porter”protein p300, as knockdown of p300 weakened the trend towards M2 polarization induced by lactic acid. Using an in vivo unilateral ureteral obstruction (UUO) mouse model, we verified the M2 polarization effect of TMAO and its detrimental role in CKD, as well as the protective effect of the TMAO inhibitor iodomethylcholine (IMC) on CKD. Clinical data validated the up-regulated TMAO’s effect on renal M2 polarization and fibrosis. Our findings suggest that CKD patients exhibit increased TMAO levels, which modulate the production of lactic acid by renal intrinsic cells. Epigenetic regulations mediated by lactic acid, particularly H4K12la on macrophage genes involved in glycometabolism, may contribute to M2 polarization. Targeting TMAO or its downstream pathways could have potential therapeutic benefits in CKD.

**Figure:**
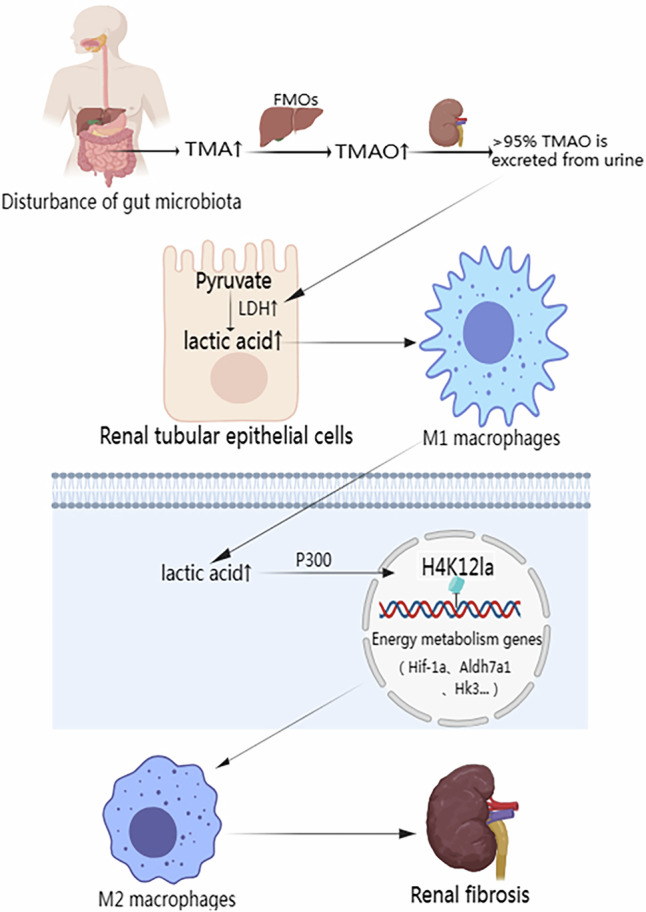
Schematic diagram showing the whole TMAO modulation process. CKD dysfunction of microbiota leads to elevated TMA. TMA metabolized through liver into TMAO which excreted 90% through kidney. Renal tubular epithelial cells contact with TMAO and secrete lactic acid affecting adjacent macrophages more into M2 type through gene histone H4K12la under the help of p300 as a carrier. These genes include a large amount of glucose metabolism related genes which could at least partially explain this M2 polarization.

Schematic diagram showing the whole TMAO modulation process. CKD dysfunction of microbiota leads to elevated TMA. TMA metabolized through liver into TMAO which excreted 90% through kidney. Renal tubular epithelial cells contact with TMAO and secrete lactic acid affecting adjacent macrophages more into M2 type through gene histone H4K12la under the help of p300 as a carrier. These genes include a large amount of glucose metabolism related genes which could at least partially explain this M2 polarization.

## Introduction

Renal fibrosis is one main chronic kidney disease (CKD) pathological process characterized by extracellular matrix (ECM) deposition in glomerular, tubulointerstitial and vascular regions [1, 2]. Although multiple diseases can lead to CKD, renal fibrosis persists throughout the entire CKD process until end-stage renal disease develops. Consequently, targeting the fibrosis process based on its underlying mechanism represents an effective therapeutic strategy for CKD.

Under certain conditions, tissue fibrosis has strong relationship with tissue macrophage subgroupsunbalance characterized as M1 and M2 polarization. This macrophage subgroups distinction was based on their preferential induction of iNOS and arginase [3]. M1 has more pro-inflammatory prone during pathogen-killing process while M2 relates more to tissue repair [4]. Macrophages M2 polarization associates tightly with renal fibrosis and CKD progression [5, 6]. M2 activation promotes renal fibrosis through multiple pathways [7–9]. Inhibiting M2 polarization could effectively reduce ECM deposition and renal interstitial fibrosis [9]. Some research suggests that the polarization of macrophages towards the M2 phenotype can be attributed to the accumulation of lactic acid. Pyruvic acid, a product of glycolysis, can be degraded through two pathways: one pathway involves lactate dehydrogenase (LDH) catalyzing the conversion of pyruvic acid to lactic acid in the cytoplasm. When this pathway is abnormally up-regulated, it leads to the accumulation of lactic acid during various pathological processes. The other pathway involves the mitochondrial pyruvate dehydrogenase complex (PDH Complex, PDC) catalyzing the conversion of mitochondrial pyruvic acid to acetyl coenzyme A (Acetyl-CoA), which is then utilized in the tricarboxylic acid cycle for energy metabolism [10]. Due to metabolic dysregulation, lactic acid accumulation occurs, resulting in specific histone lactylation on multiple genes and subsequent up-regulation of characteristic M2 genes [11, 12]. The above mechanism was approved on pulmonary fibrosis pathological model while pulmonary fibroblasts secrets large amounts of lactic acids to promote tissue macrophage histone lactylation and M2 prone gene *Arg1* expression and M2 polarization [13]. However, it remains unclear whether lactic acid accumulation also plays a role in CKD renal fibrosis, and the specific histone lactylation sites involved are still unknown.

Intestinal flora metabolism product Trimethylamine-N-oxide(TMAO) were produced from nutrient origin trimethylamine(TMA) and mainly excreted through kidney [14]. Recent research has indicated that TMAO up-regulated in CKD patients ’plasma [15] and inhibition of TMAO has been shown to down-regulate various tubulointerstitial fibrosis markers and alleviate chronic kidney injury resulting from chronic glomerulonephritis [16], the above conclusion were verified in mouse model [17]. TMAO’s pro-fibrosis effect could be partly attributed to its regulation on mitochondrial pyruvic acid and fatty acid oxidation [18] which would bring about lactic acid accumulation. Therefore, we hypothesize that during renal fibrosis, intestinal dysbiosis and inappropriate diet contribute to the accumulation of internal TMAO, which further promotes renal resident cells, such as renal tubular epithelial cells, to secrete large amounts of lactic acid, thereby influencing renal M2 polarization. This process may involve specific histone lactylation and the subsequent modulation of gene expression [12].

To elucidate the aforementioned hypothesis, we conducted experiments using mouse primary bone marrow-derived macrophages and HK-2 cells co-cultured. Our observations revealed that TMAO indeed promoted lactic acid secretion in HK-2 cells, which in turn affected macrophage M2 polarization. The E1A binding protein p300 was found to participate in this process by facilitating specific M2 gene expression.

Furthermore, validation in a unilateral ureteral obstruction (UUO) renal fibrosis mouse model confirmed the pro-fibrotic effect of TMAO. Lactylation omics and chromatin immunoprecipitation sequencing (ChIP-seq) analyses indicated that histone H4 lysine 12 (H4K12) lactylation, to some extent, accounted for the underlying mechanism. This lactylation was found on several glycolysis and oxidative phosphorylation (OXPHOS) genes. Last, we investigated the hypotheis under clinical conditions as CKD patient serum TMAO arised and related to renal fibrosis. Also, CKD renal macrophages tend to polarize into M2 polarization. The above data implies a described “TMAO’s intestinal-renal axis” and its specific molecular mechanisms contribute to TMAO’s role in CKD progression.

## Materials and methods

### Mouse bone marrow derived macrophage extraction and identification

Mice were put to death and the thigh & shinbones were extracted. The bone cavities were washed by 1640 medium and the rinsed medium were collected and centrifuged(1000 rpm, 5 min), red blood cell lysis buffer were added into the sediment and mixed for 5 min under room temperature. The cell sediments were then resuspended by 10 ml PBS and centrifuged(1000 rpm, 5 min). Remove the supernatants, add 8 ml 1640 medium(containing 10% FBS and 1%P/S), tenderly blow cells and culture the cells in 10 cm culture dishes(37 °C, 5%CO2) for 12 h. Suck out and centrifuge the unadhesive cells and culture them in 1640 medium(50 ng/ml M-CSF). Collect cells after 7 days ’ culture for subsequent trials. The above extracted cells were appraised as macrophages by FACS assay. When more than 90% cells were CD11b + F4/80 + , we consider the extraction process qualified. M1 macrophage polarization was induced by treatment with 100 ng/mL lipopolysaccharide (LPS) and 20 ng/mL recombinant mouse interferon-gamma (IFN-γ) for 12 h. Lactate intervention (10 mM) was administered. Fluorescent quantitative PCR was utilized to evaluate the expression of M1 and M2 macrophage polarization marker genes (M1 markers: iNOS, IL-12, IL-6, IL-1β, TNF-α; M2 markers: MR, Arg-1, IL-10, Fizz1, YM-1, TGF-β1). Flow cytometry was employed to assess changes in the proportions of M1 and M2 macrophages.

### Cell culture

The HK-2 cell line, derived from human renal tubular epithelial cells, was cultured in RPMI-1640 medium supplemented with 10% fetal bovine serum (FBS) at 37 °C in a 5% CO2 humidified atmosphere. Following a 48-hour incubation period, the cells were treated with 200 μmol TMAO. This intervention aimed to investigate the impact of TMAO on pyruvate metabolism and lactate production in renal tubular epithelial cells. Cell culture supernatants were collected to assess lactate levels using a lactate assay kit. Additionally, intracellular acetyl coenzyme A (CoA) levels were determined using a specific reagent kit. Protein immunoblotting and quantitative PCR analyses were conducted to evaluate the expression levels of key genes associated with pyruvate metabolism, including pyruvate dehydrogenase (PDH) and lactate dehydrogenase A (LDHA), with Histone H3 serving as an internal reference for LDHA expression.

### Real-time qPCR

For RNA extraction, 1 ml trizol was added to cells, the lysis process sustained for 5 min under room temperature. Add 200 μL chloroform and shake violently for 15 s and stand for 3 min. Centrifuge(12,000 rpm, 4 °C) for 15 min, move the supernatant to RNase-Free centrifuge tube and add isopropanol. Centrifuge the mixture(12,000 rpm, 4 °C) for 10 min and remove the supernatant, add 1 ml 75% alcohol to wash. Centrifuge (12,000 rpm, 4 °C) for 3 min, remove the supernatant and dry for 5–10 min. Add 20 μL sterile and RNase-free ddH2O to dissolve sediment. The concentration level was tested by ultraviolet spectrophotometer. For RNA reverse transcription, the reactive system(20 μl) contains 4 μl dNTP Mix, 2 μl Primer Mix, 7 μl RNA Template, 4 μl 5×RT buffer, DTT(0.1 M) 2 μL, HiFiScript(200 U/μL) 1 μl. Vortex and shake evenly, centrifuge and react under 50 °C for 50 min and 85 °C for 5 min. Centrifuge and take the cDNA for subsequent trials or −20 °C to preserve.For RT-qPCR, the primer sequences were stated in the underlying Table 1. The react system contains: 2 μl template, 1 μl primer R and 1 μl primer F, 11 μl ddH2O and 15 μl 2×sybergreen pcr master mix. The PCR amplification procedure: 95 °C for 10 min, 40 cycle(95 °C 15 s, 60 °C 30 s). The solubility curve analysis: 60–95 °C.

**Table 1:** Primers of RT-qPCR.

Test target	F	R
H-GAPDH	ACAGCCTCAAGATCATCAGC	GGTCATGAGTCCTTCCACGAT
H-LDHA	ATTCAGCCCGATTCCGTT	TGTAGCCTTTGAGTTTGATCACC
H-PDH	GTATTCCCTACTCCCTGCCACG	GGGATTCCAATTCGTCTGGGC
INOS	GTTCTCAGCCCAACAATACAAGA	GTGGACGGGTCGATGTCAC
IL-12α	TGAAGACATCACACGGGACCA	CAGCTCCCTCTTGTTGTGGAA
IL-6	GACTTCCATCCAGTTGCCTT	ATGTGTAATTAAGCCTCCGACT
IL-1β	TGAAATGCCACCTTTTGACAGT	TTCTCCACAGCCACAATGAGT
TNF-α	AGCACAGAAAGCATGATCCG	CACCCCGAAGTTCAGTAGACA
MR	TTGGACAGGGTCCTTCACTC	AACCTTCTAGTGATCTGTGGGT
ARG1	CTCCAAGCCAAAGTCCTTAGAG	AGGAGCTGTCATTAGGGACATC
IL-10	GTTCCCCTACTGTCATCCCC	AGGCAGACAAACAATACACCA
M-GAPDH	GCGACTTCAACAGCAACTCCC	CACCCTGTTGCTGTAGCCGTA
TGF-β	CTCCCGTGGCTTCTAGTGC	GCCTTAGTTTGGACAGGATCTG
FIZZ1	AACTGCCTGTGCTTACTCGT	CAAGAAGCAGGGTAAATGGGC
YM-1	ATGGAAGTTTGGACCTGCCC	CCTTGGAATGTCTTTCTCCACAG

### Western blotting

For protein extraction, using PBS to wash the cells, add 200 μl RIPA, collect cell suspend, split the cells using ultrasonication for 1.5 min. Put the cells on ice for 10 min, and centrifuge(12,000 rpm, 4 °C) for 15 min. Move the suprenatant to 1.5 ml centrifuge tube, test the extracted protein concentration by BCA kit. Samples were then boiled for 5 min, quick freezed for preparation. Electrophoresis(75 V, 130 min) till BPB moved to gel bottom. Washmambrane after Trans-membrane(300 mA),then block the mambrane for 90 min using PBST and 5% defatted milk powder. Dilute the primary antibody by 1*PBST and incubated with membrane overnight(4 °C),put the membrane back to room temperature for 30 min and washed 3 times with 1*PBST.

Primary antibody details were as follows: rabbit anti-LDHA (ab101562, 1:2000, abcam), rabbit anti-PDH (10951-1-AP, 1:2000,proteintech), rabbit anti-Histone3 (17168-1-AP, 1:3000,proteintech), rabbit anti-Collagen I (PA5-95137, 0.5 μg/ml, Thermo Fisher), rabbit anti-Fibronectin (15613-1-AP, 1:2000,proteintech), rabbit anti-Histone3 (17168-1-AP, 1:2000,proteintech), mouse anti-α-SMA (67735-1-Ig, 1:2000,proteintech), mouse anti-β-actin (66009-1-Ig 1:2000,proteintech). Secondary antibody(goat anti-rabbit IgG-HRP, ABiowell AWS0002; goat anti-mouse IgG-HRP, ABiowell AWS0001) were diluted 1:5000 and incubated with membrane for 90 min. Wash 3 times with 1*PBST and use ECL for exposure. Western blot analysis for H4k12 were done using anti-L-Lactyl-Histone H4(Lys12) Rabbit mAb(PTM-1411RM).

### Flow cytometry

Cells were cultured in a 6 cm culture dish and treated with drugs according to the experimental group. Cells were resuspended at a concentration of 1 × 10^6^ pieces/mL for staining. Each group of cells was incubated with 1 μL ofF4/80-PE (12-4801-82, eBioscience), MHC-II-APC (17-5320-82, eBioscience),CD11b-FITC (11-0112-82, eBioscience), and CD206-APC (17-2061-80, eBioscience) antibodies for 30 min at room temperature in the dark, Finally, flow cytometry was performed using a FACS Aria III (BD CANTO, USA).

### ELISA

Cell Culture supernatant werecollected by centrifuge(1000 *g*, 15 min). Add corresponding materials as diluted standard samples and tested samples to plate. Wash plate for the first time and dry it completely, add 50uL Avidin-HRP to each pore and shake evenly to incubate(37 °C, 30 min). Wash for the second time and completely dry the plate. Add chr omogenic reagent A and B and end the chromogenic reaction after 10 min. Test and analyze the OD value(Curve Expert software). The level of D-Lac were detected by a D-Lac assay Kit(H263-1-1), and the level of Ac-CoA were detected by aAc-CoA assay Kit(H331-1-1). All the kits arepurchased from Nanjing Jiancheng Bioengineering Co., Ltd. China.

### Lactylation omics detection analysis

Two groups of samples were extracted and digested, modified peptide segment were enriched, liquid chromatography-mass spectrum tandem analysis and bioinformatics analysis were done and integrated and lactation modification quantitative omics were accuratey positioned on concrete up and down regulated genes. The concrete experimental process were stated as below: (1) samples were stored at −80 °C and taken out and added x4 volume lysis buffer(1% SDS, 1% protease inhibitor, 4 μM TSA, 50 mM NAM), supersonic decomposition. 4 °C, 12,000 *g* centrifuged for 10 min, collect supernatant to new centrifuge tube, test protein concentration by BCA kit; (2) Add TCA(enternal concentration to 20%) into two samples, mixed equally, 4 °C precipiated for 2 h, 4500 *g*, centrifuge for 5 min, remove supernatant. Add precool acetone and wash 2–3 times. Dry the precipitate in the air and add TEAB (eternal concentration 200 mM), sediment were disrupted by supersonic and digested by trypsin(1:50) overnight. Use DTT to dilute into 5 mM, 56 °C to restore for 30 min. Add IAA(eternal concentration 11 mM) and incubate 15 min; (3) Dissolve peptide segment into vuffer solution (100 mM NaCl, 1 mM EDTA, 50 mM Tris-HCl, 0.5% NP-40, pH8.0), transfer supernatant into antibody purification resin(PTM Bio, PTM1404), shake tenderly overnight at 4 °C. Wash resin and elute peptide segment 3 times. Vaccum freeze dry out and demineralize(c18 ZipTips) the peptide segments for mass spectrum preparation; (4) Mobile phase A (0.1% formic acid and 2% acetonitrile) were used to dissolve peptide samples. Mobile phase B (acetonitrile-H2O containing 0.1% formic acid) were used to set different liquid gradient: 0–44 min, 6–22% B; 44–54 min, 22–30% B; 54–57 min, 30–80% B; 57–60 min, 80% B, flow velocity 450 nl/min.

Peptide were seperated by ultra efficient liquid phase system and ionized by Capillary ion source and ultimately analyzed by TimsTOF Pro mass spectrum.

### ChIP-seq

10 ng of DNA samples were prepared for Illumina sequencing as the following steps:

(1) DNA samples were blunt-ended; (2) AdA base was added to the 3’ end of each strand; (3) Illumina’s genomic adapters were ligated to the DNA fragments; (4) PCR amplification was performed to enrich ligated fragments; (5) Size selection of ~200–1500 bp enriched product using AMPure XP beads. The completed libraries were quantified by Agilent 2100 Bioanalyzer. The libraries were denatured with 0.1 M NaOH to generate single-stranded DNA molecules, captured on Illumina flow cell, amplified in situ. The libraries were then sequenced on the Illumina NovaSeq 6000 following the NovaSeq 6000 S4 Reagent Kit (300 cycles) protocol. After the sequencing platform generated the sequencing images, the stages of image analysis and base calling were performed using Off-Line Basecaller software (OLB V1.8). Sequence quality was examined using the FastQC software. After passing Solexa CHASTITY quality filter, the clean reads were aligned to Mouse genome (MM10) using BOWTIE software (V2.1.0). Aligned reads were used for peak calling of the ChIP regions using MACSV1.4.2. Statistically significant ChIP-enriched regions (peaks) were identified by comparison of IPvs Input or comparison to a Poisson background model, using ap-value threshold of 0.0001.

### UUO mouse model

Thirty male C57BL/6J mice aged 8 weeks, bred under SPF conditions and acclimatized, were randomly and blindingly divided into three groups: a blank group of 5 mice, a sham surgery group of 10 mice, and a UUO model group of 15 mice. In the UUO model group, unilateral ureteral obstruction (UUO) surgery was performed under 10% chloral hydrate anesthesia. After being anesthetized, the mice were placed in a supine position, their abdominal hair was shaved, and the area was sterilized. A 1 cm incision was made in the lower left abdomen, and the skin was sequentially cut to the peritoneum. The kidney and ureter were dissected, and the midsection of the left ureter was lifted with tissue forceps. The ureter was ligated with silk thread at both ends and then cut, followed by continuous skin suturing. The mice in the sham surgery group underwent the same procedure under anesthesia, but without ligation of the ureter. The blank group received no treatment. After surgery, the mice in the UUO model group were further divided into a normal drinking water group of 5 mice, a TMAO intervention drinking water group of 5 mice (with 0.3% TMAO(1184-78-7, APExBIO) in the drinking water), and a TMAO inhibitor (IMC(624-95-3,Abiowell), 0.06% in drinking water) group of 5 mice. The mice in the sham surgery group were divided into a normal drinking water group of 5 mice and a TMAO intervention drinking water group of 5 mice (with 0.3% TMAO in the drinking water). This intervention continued for 14 days.

### CKD patient inclusion and exclusion criteria

We collected data from CKD patients with clear diagnosis. All 30 patients were 18–70 yrs old with renal biopsy results as primary glomerulonephritis. All had the ability to communicate and had no cognitive or mental disorder. All CKD patients provided ingformed consent and voluntarily participated. We excluded secondary nephropathy patients, history of inflammatory diseases and malignant as well as benign tumors patients, history of liver and gastrointestinal disease patients, history of kidney transplantation patients, patients with history of taking probiotics, antibiotics, non-steroidal anti-inflammatory drugs, metformin, vitamins, steroids or immuno-suppressive drugs within one month before inclusion in the study. Special diets patients(such as consuming red meat, seafood, egg yolks five times or more a week, as well as being a vegetarian or having a pure meat diet), history of enteral nutrition or parenteral nutrition patients, pregnant or lactating women were also excluded.

### Clinical data collection

It includes indicators such as patients’ age, gender, 24-h urinary protein (24 h-utp), estimated glomerular filtration rate(eGFR), serum creatinine, blood urea nitrogen, uric acid, triglyceride, total cholesterol, complement C3, C4, Immunoglubulin IgA, IgG, IgM, etc. All the clinically relevant test indicators were completed in the clinical laboratory, biochemical laboratory and nephrology specialized laboratory of the 3^rd^ Xiangya Hospital of Central South University.

### Determination of plasma TMAO

Approximately 4–5 ml blood specimens were collected from CKD patients undergoing renal biosy and healthy controls. The samples were centrifuged and supernatants were collected for TMAO detection: (1) Set up standard sample wells and sample wells. Add 50 μL of standard samples with different solubility values to each standard sample well; (2) Set up a blank well and sample wells to be tested. First, add 10 μL of sample diluent to the sample wells of the microplate, and then add 40 μL of the sample to be tested. (3) Enzyme Addition: Except for the blank well, add 100 μL of enzyme-labeled reagent to each well; (4) Incubation: After sealing the plate, incubate at 37 °C for 60 min; (5) Dilute the 20-fold concentrated washing solution 20 times with distilled water for later use; (6) Washing: Remove the sealing film, discard the liquid, and shake dry. Fill each well with the washing solution, let it stand for 60 s, discard the liquid, repeat 5 times, and pat dry; (7) Color Development: Add 50 μL of color developing agent A and 50 μL of color developing agent B to each well in sequence, gently shake to mix evenly, and develop color in the dark at 37 °C for 15 min; (8) Termination: Add 50 μL of terminator to each well to terminate the reaction; (9) Measurement: Use the blank well to zero, measure the absorbance (OD value) of each well at a wavelength of 450 nm, and complete the measurement within 15 min after adding the terminator; (10) Take the concentration of the standard sample as the ordinate and the OD value as the abscissa, analyze it with the professional curve-making software “Curve Expert”, and make a standard curve according to the prompts. Calculate the regression equation of the standard curve based on the concentration of the standard sample and the OD value, substitute the OD value of the sample into the equation, and calculate the sample concentration.

## Renal pathological index determination

### Masson staining

Bake the slices at 60 °C for 12 h. Dewax the slices to water, shake off water, then drop nuclear staining solution to cover the entire tissue for 30 s. Rinsing off staining solution with distilled water, soak the slices in PBS to make the cell nuclei turn blue. Shake off PBS, drop cytoplasm staining solution to cover the entire tissue, stain for 3 min, rinse off. Use color seperation solution for color seperation for 30 s and discard. Drop counterstaining solution to cover the entire tissue, discard after 5 min and rinse. Blow dry and cold setting, make slices transparent with xylene, and seal the slice. After which scan and image with a panoramic scanner.

### Immunohistochemistry

Bake the slices at 60 °C for 12 h. Dewax the slices to water. Antigen retrieval by heat. Add 1% periodic acid and inactivate the endogenous enzymes at room temperature for 15 min. Wash with pbs for 3 times. Incubate with primary antibody(Col I Abiowell, AWA59148, α-SMA Abiowell, AWA10574) with a dilution ratio to 1:200 and incubate overnight. After washing, incubate with the secondary antibody(Rabbit) 50 to 100ul-IgG antibody-HRP polymer and incubate at 37 °C for 30 min. Wash and develop DAB color. Counterstain with hemotoxylin for 5–10 min, rinse and dehydrate with alcohol of various concentrations for 5 min at each level. Take out and place in xylene for 10 min, twice, seal with neutral gum, and observe under a microscope, scan and image with a panoramic scanner.

### Immunofluorescence

Bake the slices at 60 °C for 12 h. Dewax the slices to water. Antigen retrieval by heat. Place the slices in a sodium borohydride solution at room temperature for 30 min, rinse for 5 min. Block endogenous oxides with 0.3% hydrogen peroxide and block with 10% normal serum. Incubate with the primary antibody(CD80) with a dilution ratio of 1:100 and incubate overnight. Rinse and incubate with immunohistochemistry HRP secondary antibody for 0.5 h and rinse. Incubate with TYP-520 fluorescent dye at 37 °C as fluorescent dye reation and rinse. Antigen retrieval by heat and block endogenous oxides. Block with 10% normal serum, incubate with the primary antibody (CD163) with a primary antibody dilution ratio of 1:100, incubate at 4 °C overnight. Rinse with PBS and incubate with immunohistochemistry HRP secondary antibody. Incubate at 37 °C for 30 min, and rinse with PBS. Incubate with TYP-620 fluorescent dye at 37 °C in the dark, rinse and stain the nuclei with DAPI working solution and rinse. Seal the slices with buffered glycerol. Store in the dark. Scan and image with a panoramic scanner. The image analysis software used is Image-Pro-Plus 6.0.

### Statistic analysis

The research data were presented as mean ± SD, and all statistical analyses were conducted using SPSS 20.0 software (SPSS, IBM, Armonk, NY, USA). A one-way analysis of variance (ANOVA) was employed to compare between the groups. Statistical significance was considered when *P* < 0.05. Throughout the entire experiment, data analysis was carried out by investigators blinded to the group identities. Clinical data were analyzed by GraphPad Prism 8. Shapiro-Wilk were used to for normality testing. For measurement data that follow a normal distribution, they were expressed as mean ± standard deviation. For measurement data do not follow a normal distribution, they were expressed as median (P25, P75). Count data were expressed as a percentage(%). An independent samples t-test was used for normal distribution data. For categorical variables, a chi-square test was used. Pearson and spearman analysis was used for correlation analysis depending on whether data was normal or non-normal distribution. P value less than 0.05 indicate that the difference was statistically significant.

## Results

### TMAO up-regulates UUO mice renal fibrosis and renal macrophage M2 polarization

**Fig. 1:**
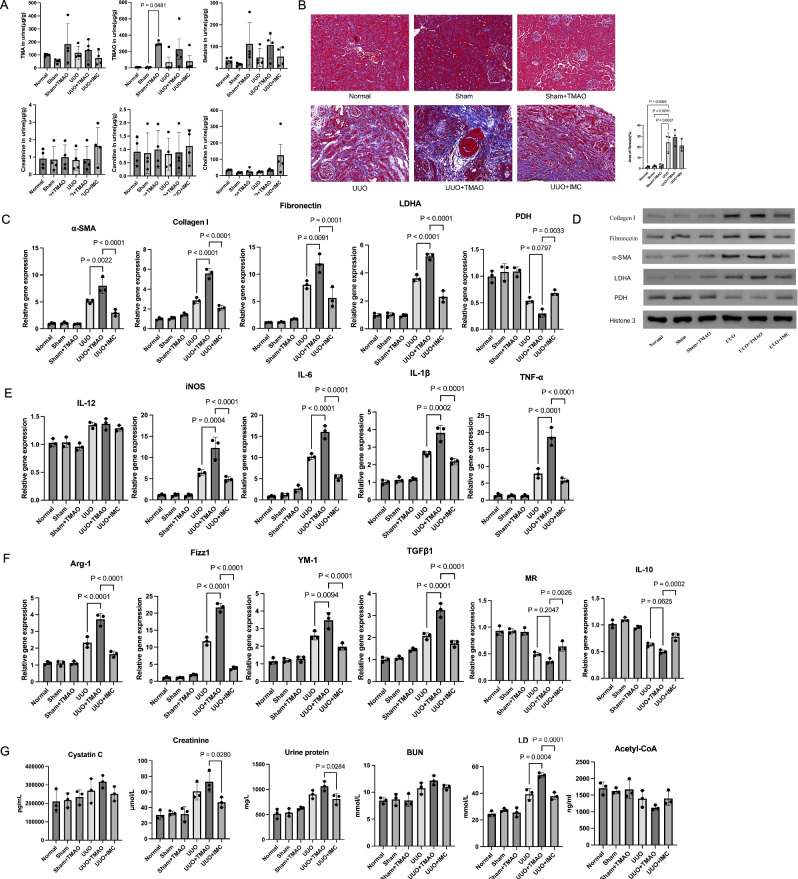
TMAO exacerbates renal fibrosis in UUO mice. In view of the high consistency among the same mouse species, 30 mice were divided into 6 groups, each consisting of 5 mice. The Normal group received no treatment, the Sham group underwent a laparotomy procedure, the Sham+TMAO group underwent the laparotomy procedure and received drinking water containing 0.3% TMAO, the UUO group underwent left kidney ureteral ligation, the UUO + TMAO group underwent left kidney ureteral ligation and received drinking water containing 0.3% TMAO, and the UUO + IMC group underwent left kidney ureteral ligation and received drinking water containing the TMAO inhibitor IMC at 0.06%, the intervention continued for 14 days. **A** The level of TMA, TMAO, Betaine, Creatinine, Carnitine, Choline in urine of different groups of mice. **B** Masson stain of the left kidney of different groups of mice. **C** α-SMA, collagen I, fibronectin, LDHA, PDH mRNA expression in the left kidney of different groups of mice. **D** Western blotting showing the α-SMA, collagen I, fibronectin, LDHA, PDH levels of the left kidney of different groups of mice. **E** IL-12, iNOS, IL-6, IL-1β, and TNF-α mRNA expression in the left kidney of different groups of mice. **F** Arg-1, Fizz1, YM-1, TGFβ1, MR, IL-10 mRNA expression in the left kidney of different groups of mice. **G** Elisa showing the Cystatin C, Creatinine, BUN, LD, Acetyl-CoA level in the serum, and the urine protein.

Trimethylamine-N-oxide (TMAO) is auremic toxin, which has been associated with CKD. Renal infiltration of inflammatory cells including macrophages is a crucial event in kidney fibrogenesis. However, how macrophage regulates fibroblast activation in the fibrotic kidney remains elusive. We found that in UUO mice, compared to sham surgery mice and normal mice, there was an increase in TMAO levels in the urine(Fig. 1A), but no significance change in faces (Supplementary Fig 1).

However, upon examining the levels of α-SMA, collagen I, and fibronectin in renal tissue (Fig. 1C), we observed a significant increase in renal fibrosis in UUO mice following TMAO intervention, which was effectively reversed by IMC treatment (Fig. 1B). Additionally, the levels of LDHA in the kidney displayed similar changes, while PDH showed an opposite trend (Fig. 1C). WB also shown the same (Fig. 1D, wb full vision in supplementary Fig 4). We also evaluated markers associated with macrophage polarization in the kidney and found that, except for IL-12, the levels of iNOS, IL-6, IL-1β, and TNF-α, which are specific markers for M1 macrophages, significantly increased after TMAO intervention and decreased after IMC inhibition of TMAO (Fig. 1E). Conversely, among markers specific for M2 macrophages, including Arg-1, Fizz1, YM-1, and TGF-β1, their levels also showed a significant increase after TMAO intervention and a decrease after IMC inhibition of TMAO (Fig. 1F). However, the trends of MR and IL-10 were opposite. This may be attributed to the fact that these markers were detected in renal tissue rather than specifically in renal macrophages.

In serum, the levels of Cystatin C, Creatinine, BUN, and LD all increased after TMAO intervention and decreased after IMC inhibition of TMAO. However, Acetyl-CoA itself was observed to decrease in UUO mice (Fig. 1G). Meanwhile, we detected the polarization of macrophages in the kidney, the MHCII + M1 decreased in the UUO mice intervented with TMAO (Supplementary Fig. 2A), and the CD206 + M2 increased (Supplementary Fig. 2B).

### TMAO potentiates macrophage M2 polarization when co-cultured with HK-2

To mimic renal tubular environment, we co-cultured macrophages and HK-2 cells with or without TMAO to evaluate TMAO’s effect on M1/M2 polarization.

**Fig. 2:**
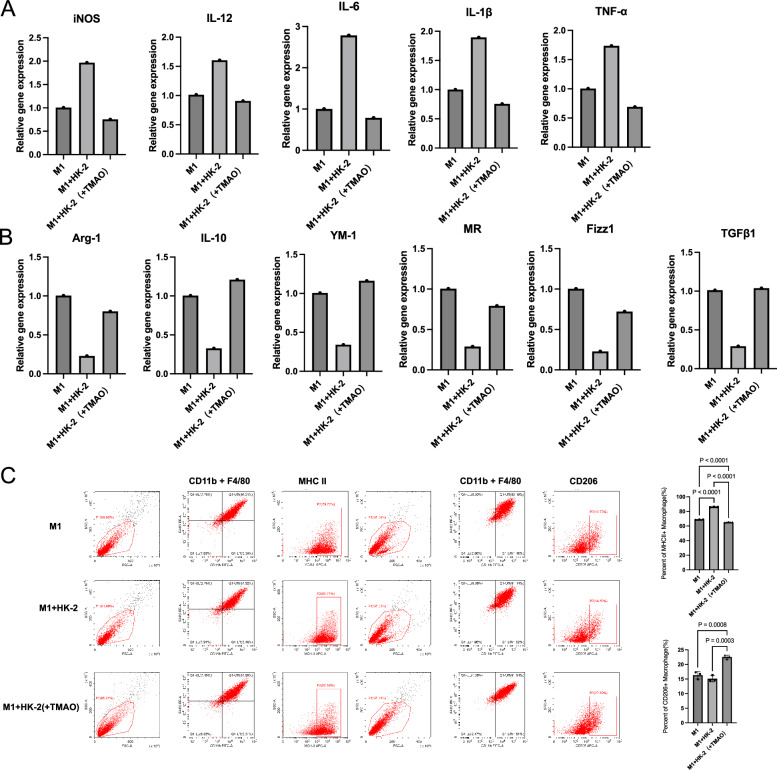
TMAO enhances M2 polarization in macrophages co-cultured with HK-2 cells. **A** iNOS, IL-12, IL-6, IL-1β, and TNF-α mRNA expression in M1, M1 cocultured with HK-2 for 48 h, HK-2 treated with TMAO(200 μM) for 48 h then cocultured with M1 for 48 h. **B** Arg-1, IL-10, YM-1, MR, Fizz1, and TGFβ1 mRNA expression in M1, M1 cocultured with HK-2 for 48 h, HK-2 treated with TMAO(200 μM) for 48 h then cocultured with M1 for 48 h. **C** Flow cytometry plots and quantification of MHCII+ macrophage(M1) and CD206+ macrophage(M2) in M1, M1 cocultured with HK-2 for 48 h, HK-2 treated with TMAO(200 μM) for 48 h then cocultured with M1 for 48 h.

M1-related markers such as iNOS, IL-12, IL-6, IL-1β, and TNF-α all increased after co-culture with M1 macrophages and HK-2 cells (Fig. 2A). However, when TMAO was added during co-culture, these markers significantly decreased. Conversely, M2 macrophage-related markers including Arg-1, IL-10, YM-1, MR, Fizz1, and TGF-β1 decreased after co-culture with M1 macrophages and HK-2 cells, but increased when TMAO was added (Fig. 2B). This suggests that co-culture with HK-2 cells enhances the polarization of M1 macrophages, but TMAO treatment during co-culture promotes the transition of M1 macrophages towards the M2 phenotype. Flow cytometry appraisal results showed TMAO addition truly raised CD206 + M2% (Fig. 2C).

### TMAO regulates HK-2 cells ’ pyruvate metabolism which caused lactic acid accumulation

HK-2 cells secretion could be changed by TMAO regulation which affected renal macrophage surrounding environment. To further explore how TMAO regulates HK-2 cells ’ metabolism condition, two key enzymes in pyruvate’s degradation: LDHA and PDH levels were measured by WB and PCR (Supplementary Fig. 3A). Pyruvate degradation balance were broken when LDHA up-regulated and PDH down-regulated after TMAO (200 µmol) 48 h treatment. Therefore, pyruvate degradation product LD (LDHA catalyzed) accumulated and acetyl-CoA reduced in HK-2 cells(Supplementary Fig. 3B).

### Macrophage polarization under lactic acid modulation simulates TMAO regulation

**Fig. 3:**
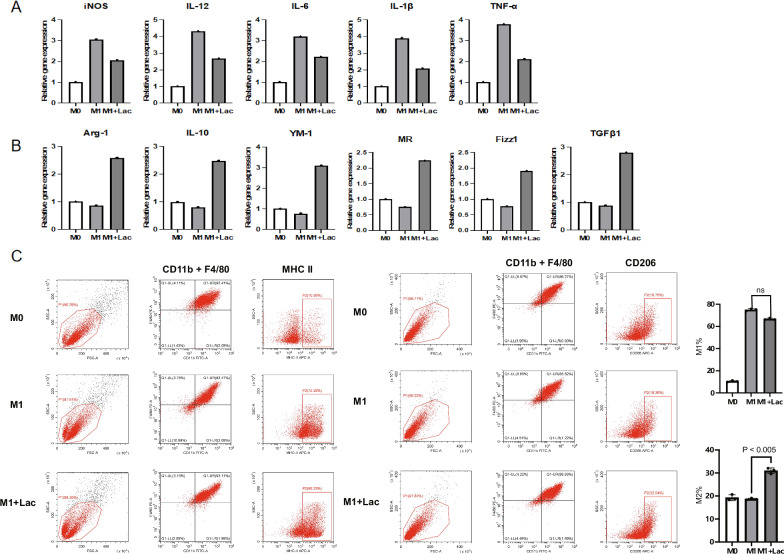
Macrophage polarization under lactic acid modulation simulates TMAO regulation. **A** qRT-PCR was developed on macrophages treated under certain conditions as elucidated on Method part, M0, M1(100 ng/ml LPS + 20 ng/ml IFN-γ for 12 h) and M1 + LD 10 mM, iNOS, IL-12, IL-6, IL-1β, and TNF-α mRNA levels (**A**) and Arg-1, IL-10, YM-1, MR, Fizz1, and TGFβ1 levels (**B**) were shown and Flow cytometry plots and quantification of MHCII + M1 and CD206 + M2 were shown (**C**).

Lactic acid(LD)(10 mM) intervention distinctly changed M1/M2 balance while M2 polarization occurred. The effect in some degree was similar to TMAO regulated HK-2’s affection on macrophage. M1 and M2 characteristic gene profiles mentioned above were up or down-regulated according to M2 polarization (Fig. 3A, B). MHCII + M1 decreased with the intervention of LD, and the CD206 + M2 increased (Fig. 3C).

These results pointed out that in TMAO’s pro-M2 polarization process, lactic acid accumulation was undoubtedly the key and adequate condition.

### p300 should be key lactic nuclear messenger which regulates M2 gene expression

**Fig. 4:**
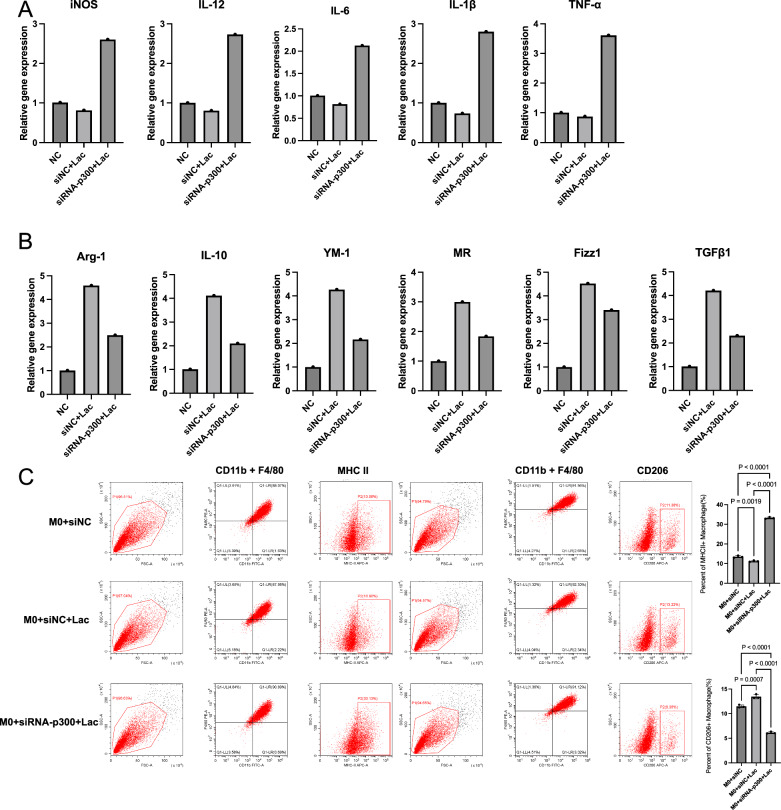
p300 is a crucial nuclear mediator of lactate signaling that regulates the polarization of M2. **A** iNOS, IL-12, IL-6, IL-1β, and TNF-α mRNA expression in BMDM transfected with siNC, BMDM transfected with siNC and treated with 10 mM lactate for 48 h, BMDM transfected with siRNA-p300 and treated with 10 mM lactate for 48 h. **B** Arg-1, IL-10, YM-1, MR, Fizz1, and TGFβ1 mRNA expression in BMDM transfected with siNC, BMDM transfected with siNC and treated with 10 mM lactate for 48 h, BMDM transfected with siRNA-p300 and treated with 10 mM lactate for 48 h. **C** Flow cytometry plots and quantification of MHCII+ macrophage(M1) and CD206+ macrophage(M2) in BMDM transfected with siNC, BMDM transfected with siNC and treated with 10 mM lactate for 48 h, BMDM transfected with siRNA-p300 and treated with 10 mM lactate for 48 h.

To test whether p300 send lactic acid accumulation message to the nucleus, we knocked down cellular p300. M1 and M2 characteristic gene expression varied respectively (Fig. 4A, B). And observed the lactic acid’s pro-M2 polarization effect weakened (Fig. 4C). These results manifests p300’s vital role as a messenger in lactylation-regulated M2 polarization.

### Lactylation omics detection analysis positioned histone H4K12la as M2 effect mediator

**Fig. 5:**
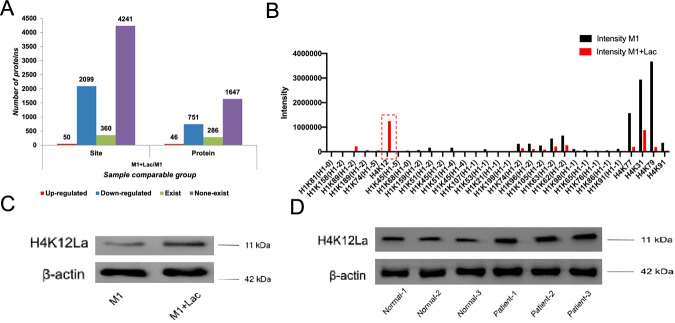
Lactylation of histone H4K12 promotes polarization of macrophages towards the M2 phenotype. **A** The number of lactylation modification sites and proteins, as well as the quantity change between presence and absence of lactylation modification in M1 or M1 treated with 10 mM lactate for 48 h. **B** Changes in histone modification sites and their intensity between the two groups. **C** Western blotting showing the H4K12La in the two groups. **D** Western blotting showing the H4K12La in the macrophages in peripheral blood of the healthy people and CKD patients.

To investigate the lactylation modification of macrophages under lactate intervention. Mice bone marrow derived macrophages were divided into two groups: M1(100 ng/ml LPS and 20 ng/mlIFN-γ) and M1 with lactic acid (10 nM). Two groups of macrophages proteins were extracted and quantitative omics of lactate modification were tested. After the addition of lactate (Lac), there were significant changes in lactylation modification sites and proteins in macrophages (Fig. 5A), with histone H4 K12 showing the most significant increase (Fig. 5B). We validated this result through WB analysis (Fig. 5C, wb full vision in supplementary Fig. 4) and found that compared to healthy individuals, macrophages in the peripheral blood of patients with renal fibrosis exhibited a significant increase in lactylation at the H4K12 site (Fig. 5D, wb full vision in supplementary Fig 4).

### Glycolysis associated genes may serve as the key candidate genes associated with H4K12la

**Fig. 6:**
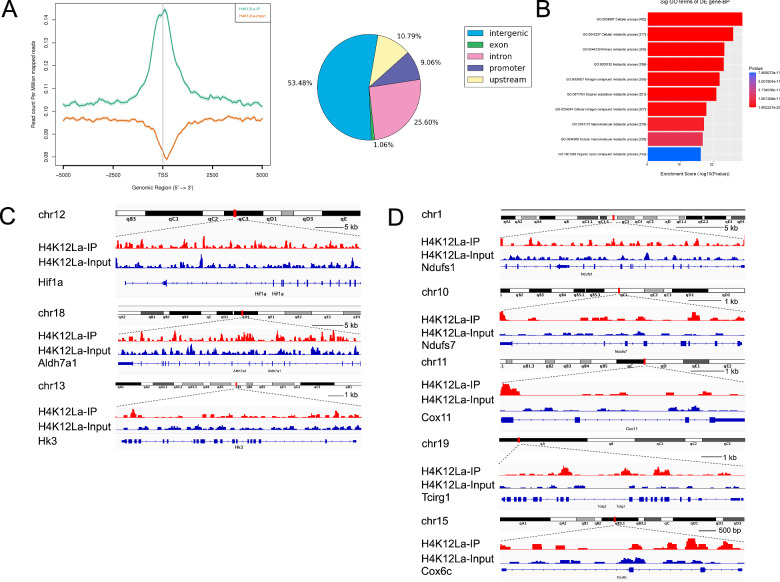
H4K12La initiates metabolic related genes expression in macrophages. **A** Distribution of H4K12La sites relative to translation start site (TSS) and the portion of site with lactylation. **B** GO analysis of H4K12La peaks. **C** Normalized read densities for H4K12La at the Hif-1α, Aldh7a1, Hk3 genes. **D** Normalized read densities for H4K12La at the Ndufs1, Ndufs7, Cox11, Tcirg1, Cox6c genes.

To uncover the regulatory role of histone lactylation in gene expression, we first performed chromatin immunoprecipitation followed by sequencing (ChIP-seq) using anti-H4K12la antibodies. Our ChIP-seq data showed that H4K12la was enriched in promoter regions (Fig. 6A). AGO analysis revealed that these H4K12la-specific genes were enriched in metabolic pathways, suggesting a regulatory effect of histone lactylation in macrophages (Fig. 6B). In addition, H4K12la peaks were validated at the promoters of the glycolysis associated genes (*Hif-1α,Aldh7a1, Hk3*) (Fig. 6C), and OXPHOS associated genes(*Ndufs1*, *Ndufs7, Cox11*, *Tcirg1*, *Cox6c*) (Fig. 6D). In summary, we found that TMAO in urine leads to the secretion of lactate by renal tubular epithelial cells. Lactate, in turn, induces lactylation of H4K12 in renal macrophages, resulting in polarization towards the M2 phenotype and exacerbating renal tissue fibrosis(Graphical Abstract).

### Serum TMAO level were up-regulated and positively associated with renal fibrosis and renal M2 polarization

**Fig. 7:**
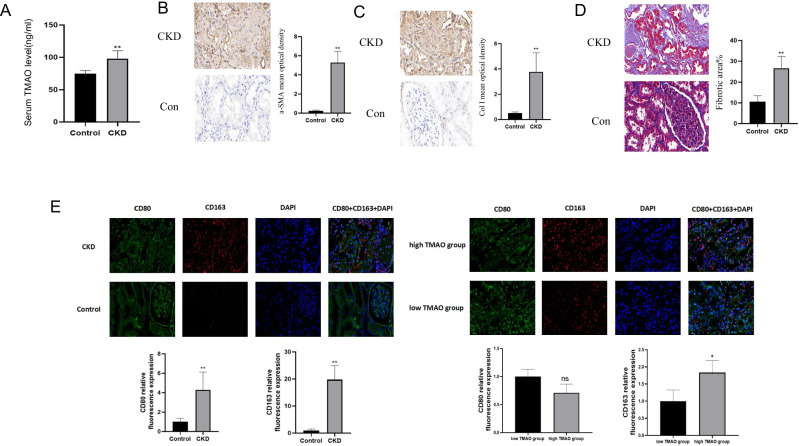
Serum TMAO level were up-regulated and positively associated with renal fibrosis and renal M2 polarization. **A** 30 CKD patients mean serum TMAO level is statistically higher compared to 12 control(6 renal tumor relatively normal resected tissue and 6 healthy renal tissue), **p < 0.01. **B**–**D** CKD renal tissue α-SMA, Collegen I immunohistochemistry images (x50) and fibrotic area% (Masson staining) compared to control (renal tumor resected relatively normal tissue), **p < 0.01. **E** Immunofluorescence images(x50) on 6 CKD and 6 Con(renal resected) CD80(M1) and CD163(M2), *p < 0.05.

In order to verify whether our theory can be consistent with clinical practice, we conducted a clinical study. The results showed that CKD patients’ serum TMAO levels were indeed up-regulated compared to those in the control group (Fig. 7A). To investigate the relationship between the up-regulated serum TMAO and clinical indicators of CKD, we first validated CKD renal fibrosis tendency (Fig. 7B–D). Then we statistically analyzed the relevant clinical data and the results suggested that CKD patients with higher serum TMAO levels tend to have more serious renal fibrosis and higher 24 h-utp(an independent risk factor for CKD progression) (Table 2). In addition, there was also a statistically significant positive correlation between serum TMAO and M2 trend in renal tissue (Fig. 7E).

**Table 2:** High and low serum TMAO groups’ comparison on general conditions, biochemical indicators and renal fibrosis related indicators.

Variables	Low TMAO group	High TMAO group	*P* value
	*N* = 15	*N* = 15	
Age	34.87 ± 11.98	38.00 ± 12.93	0.49
Male (*n*,%)	5 (33)	8 (53)	0.46
24 h urinary protein	971 (440–1486)	1440 (1026–1732)	0.04*
eGFR	93.58 ± 27.80	81.86 ± 31.9	0.29
Serum Creatinine	82.20 ± 27.42	98.80 ± 33.52	0.14
Blood Urine Nitrogen	5.50 ± 2.12	6.77 ± 2.6	0.16
Serum Uric Acid	349.5 ± 129.4	359 ± 93.9	0.82
Triglyceride	1.647 ± 0.77	1.528 ± 0.55	0.63
Total cholesterol	4.19 (3.82–5.01)	4.50 (4.11–5.11)	0.42
Complement C3	0.94 ± 0.17	0.86 ± 0.14	0.19
Complement C4	0.23 (0.18–0.27)	0.26 ± 0.09	0.28
IgA	2.59 ± 0.85	2.93 ± 0.75	0.26
IgG	12.53 ± 2.93	11.28 ± 2.43	0.21
IgM	1.35 ± 0.36	1.49 ± 0.41	0.36
Serum TMAO level	87.97 ± 7.121	108.3 ± 6.321	<0.01
Renal a-SMA	4.88 ± 0.92	5.71 ± 1.24	0.04*
Renal Col I	3.42 ± 1.28	4.11 ± 1.67	0.02*
Renal fibrotic area%	24.15 ± 3.80	29.10 ± 6.07	0.01*

*p < 0.05, comparison between two groups has statistical significance.

## Discussion

We put forward and preliminarily verified through animal experiments the following conclusion: In CKD, there exists an imbalance in the gut microbiota. TMAO, a product of the gut microbiota, reaches the renal microenvironment via the bloodstream and prompts renal intrinsic cells, such as renal tubular epithelial cells, to secrete an excessive amount of lactic acid. The high concentration of lactic acid in the local renal environment induces macrophages infiltrating the kidney to tend towards M2 polarization, thereby aggravating renal fibrosis. The specific molecular mechanisms may involve the regulation of the transcription of various genes, including those associated with glycolysis and oxidative phosphorylation, through lactylation facilitated by the acetyltransferase p300. The disruption of energy metabolism in macrophages is likely to be a crucial factor contributing to their M2 polarization since we validated this trend on clinical data.

Gut microbiota products have been found to be strongly associated with CKD outcomes [19]. CKD patients harbor distinct microbial species due to toxin accumulation, resulting in differences in intestinal microbial products compared to healthy individuals. The kidneys are primarily responsible for excreting most of these bacterial products, including TMAO [20]. As previously mentioned, TMAO has been implicated in promoting CKD progression and adverse outcomes based on numerous clinical investigations and animal studies. TMAO regulates the immune microenvironment in the kidneys, particularly macrophage polarization [21, 22]. Direct stimulation by TMAO has been shown in several studies to induce M1 polarization, thereby triggering inflammation. However, M2 macrophages are believed to play amore significant role in CKD-related chronic fibrosis. Our research first suggests the possibility that TMAO indirectly promotes M2 polarization by directly modulating the metabolism of renal resident cells, such as renal tubular epithelial cells.

Except TMAO, other uremic toxins gut microbiota may produce includes p-cresyl sulfate(PCS) and indoxyl sulfate(IS). As CKD renal function declines, the concentration of these uremic toxins showed an upward trend [23, 24] which had a strong relationship with CKD progression [25]. PCS binds to albumin and accumulated within cells which leads to nephrotoxicity. It increases the activity of NADPH oxidase and the production of ROS, thereby inducing the expression of inflammatory cytokines associated with renal fibrosis [26]. Human renal proximal tubular cells treated with IS will exhibit typical morphological changes of apoptosis, upregulating the expression of the pro-apoptotic protein bax, and disrupting the mitochondrial metabolic function [27]. Besides, IS interferes with the oxygen metabolism of renal tubular cells through oxidative stress, exacerbating renal dysfunction [28].

Probiotics are active microorganisms beneficial to health of the host, maining exerting their effects by improving the balance of the intestinal flora. Currently, the probiotics that have been studied more include Saccharomyces cerevisiae, Streptococcus genus, Lactobacillus genus and Bifidobacterium genus [29, 30]. In a mouse model of CKD induced by ischemia-reperfusion injury, supplementation of the probiotic Lacticaseibacillus casei Zhang could decrease the levels of serum creatine and blood urea nitrogen, relieve renal fibrosis, improve intestinal flora dysbiosis, elevate the level of short-chain fatty acids and nicotinamide, and regulate the immune response [31]. In adenine-induced CKD mouse model, the use of probiotics containing a mixture of lactic acid bacteria strains can not only alleviate kidney damage and protein associated with fibrosis, enhance the renal immune response, but also reverse the dysbiosis of the intestinal flora, restore the abundance of symbiotic bacteria, and improve the integrity of the intestinal barrier [32]. The benefit of probiotics in delaying CKD progression has also been observe in human population: a randomized controlled trial conducted on CKD 3a patients has shown that compared with the placebo group, the probiotic group has an increased abundance of Lactobacillales and Bifidobacterium in feces, and better iron metabolism status, parathyroid hormone, and β2 microglobulin, and it may bring certain benefits to cardiovascular outcomes [33]; After intervention with probiotic Lacticaseibacillus casei Zhang, CKD3-5 stage patients significantly decreased with their serum Cystatin C and parathyroid hormone, the albumin-to-creatinine ratio in urine dropped too, and finally the decline rate of estimated glomerular filtration rate(eGFR) [31]. The above result all indicate that probiotic supplementation has the potential to slow down CKD progression.

Further investigation revealed that lactic acid secreted by these resident cells contributes, at least to some extent, to the polarization of adjacent macrophages towards the M2 phenotype. Although other secreted molecules may also contribute to M2 polarization, our study indicates that lactic acid has the potential to induce this M2 tendency on its own. We speculate that the underlying mechanism involves the reprogramming of macrophage metabolism under specific lactic acid concentrations. Lactic acid has been shown to modulate the metabolic conditions of immune cells.

High environmental concentrations of lactic acid inhibit the glycolysis of immune cells and increase oxygen consumption [34]. This phenomenon also occurs under inflammatory conditions to protect macrophages from over-activation as a compensatory mechanism [35]. In other words, lactic acid, as a signaling transducer to some extent, regulates both physiological and pathological metabolism [36].

Extracellular and intracellular lactic acid, with varying concentrations, transduce signals that exert diverse functions [37]. One important mechanism by which lactic acid regulates macrophages is through histone translational modification, specifically histone lysine lactylation [38]. However, the nuclear import of lactic acid and the precise localization of histone lysine require operational backup mechanisms. As a paralog of CREB, p300 (a member of the HAT family) is involved in the process of gene lactylation and regulates the expression of specific genes [13]. Under pathological conditions, p300 serves as an essential transducer or “writer” in the histone lysine lactylation of phagocytes [39, 40]. We hypothesized that p300 is also necessary in CKD lactic acid-modulated macrophage models, and subsequent experiments confirmed our hypothesis: inhibition of p300 attenuated this M2 trend.

However, the nuclear modulation steps of lactic acid-p300 and the localization of target genes require further investigation through experiments. This could pave the way for a promising pathway to alleviate CKD fibrosis. Additionally, other lactylation enzymes may also play roles in this process besides p300, which warrants further investigation.

Histone lactylation has been recognized as an vital epigenetic factor contributing to kidney diseases. For instance, H3K14la/KLF5 drives epithelial-mesenchymal transition which is widely recognized as a critical contributor to diabetic kidney disease [41]. Besides, in sepsis-associated acute kidney injury, H3K18la is elevated which lead to RhoA/Rho-associated protein kinase (ROCK)/Ezrin signaling, activation of NF-ｋB, inflammation, cell apoptosis, and aggravated renal dysfunction [42]. However, there still lack research on immune cells histone lactylation as monocytes and macrophages under renal disease conditions.

Since lactylation on a panel of histone lysines regulates macrophage conditions and prevents macrophage over-activation [10], we next investigated the specific histone types and lysine sites involved in CKD progression through lactylation omics. The most significantly up-regulated lactylation sites were found to be on H4K12,which is associated with several glycolysis and oxidative phosphorylation genes. H4K12la has previously been reported as a central linkin the activation of microglial glycolysis in Alzheimer’s disease [41]. In our study, lactate from glycolysis and pyruvate metabolism was associated with glycolysis genes such as*Pkm2*, *Hif-1α*, and *Ldha*.

These lactylations positively feedback glycolysis and microglial activation. However, our research yielded an unexpected result: H4K12la was associated with an M2 trend, while glycolysis was not significantly activated. This disparity could be attributed to differences in disease models and pathological environments. Additionally, our research uncovered that H4K12la is present on several oxidative phosphorylation gene promoters, including *Ndufs1*, *Ndufs7*, *Cox11*, *Tcirg1*, and *Cox6c*, in addition to glycolysis gene promoters such as *Aldh7a1* and *Hk3*. Lactylation on these genes as an epigenetic regulation mechanism has been rarely reported. Simultaneous lactylation on a panel of genes may have unpredictable effects, as it represents a comprehensive result. To assess the impact of these targeted genes on kidney disease progression and fibrosis, we provided an overview of several genes and offered a preliminary assessment of whether they are “protective” or “damaging” in CKD.

Ndufs1 serves as the largest core subunit of mitochondria complex I, which plays a pivotal role in electron transportation and NADH oxidation [40]. Additionally, Ndufs1 has been shown to inhibit the production of reactive oxygen species (ROS), which are byproducts of ATP production [42, 43]. Furthermore, a separate study demonstrated that Ndufs1 has therapeutic potential in alleviating cardiac dysfunction and myocardial fibrosis following myocardial infarction [44]. Collectively, Ndufs1 appears to have a “protective” role against tissue damage. However, its specific role in CKD orAKI has not been extensively investigated. The epigenetic regulation of Ndufs1 by H4K12la may represent a promising pharmacological target for further research in CKD.

Cytochrome c oxidase Cox6c is a transmembrane protein located in the mitochondria and is involved in the respiratory electron-transport chain. Cox6c levels have been found to be down-regulated in hemodialysis patients, and this decrease is negatively correlated with malondialdehyde levels [45]. Similarly, peritoneal dialysis patients also exhibit lower levels of Cox6c in peripheral blood mononuclear cells [46]. These findings suggest a potential association between Cox6c and the late-stage pathological processes of renal dysfunction.

Hif-1α is a subunit of the heterodimer Hif-1. Under hypoxic conditions, Hif-1α is stabilized, leading to the activation of total nuclear Hif-1, which regulates the expression of multiple genes [47]. Hif-1α has been studied for its protective role in renal ischemia/reperfusion injury (RIRI) [48, 49], as well as its tight association with CKD [50]. In our study, we detected H4K12la on Hif-1α genes, although not on gene promoters, which may have an unknown effect on its expression or function.

Exploring this association could be a meaningful direction for CKD research, but further validation is needed, as H4K12la has been reported to target Hif-1α, with downstream effects on glycolysis [51].

Hexokinase 3 (HK3) is an enzyme involved in the phosphorylation of glucose and has been considered a diagnostic marker for idiopathic pulmonary fibrosis disease [52]. In certain acute autoinflammatory conditions, such as autoimmune thyroid diseases, HK3 has been identified as a key metabolic gene driving the M1 trend [53]. However, its role in CKD fibrosis and the M2 trend has not yet been reported. Investigating the involvement of HK3 in these processes could provide valuable insights into the pathogenesis of CKD fibrosis.

Finally, we validated the aforementioned mechanism and assessed the pharmacological potential of the TMAO inhibitor iodomethylcholine (IMC) in vivo. IMC, a choline analog, inhibits TMAlyase activity, thereby reducing plasma TMAO levels [54]. Studies have shown that IMC alters the gut microbiota composition to favor “good bacteria” and alleviates hypertension in CKD mice [55]. Consistent with these findings, our results demonstrated that IMC reduced the area of renal fibrosis and improved proteinuria and renal function. Interestingly, fecal TMAO levels did not show significant changes following IMC treatment, possibly due to TMAO excretion through the kidneys. Therefore, fecal TMAO levels may not be a precise indicator for evaluating CKD clinically.

Our research still has its own limitations which we summarized as following points: first, most of the results were based on animal model and cells of animal origin, clinical data should be added next to validate the hypothesis and reflect its clinical application value; second, since various glycolysis genes and non-glycolysis genes have been sorted out by ChIP-seq, validation tests should be added next such as ChIP-qpcr to make sure these genes really were regulated by lactylation, even we should sort out the most important glycolysis gene or genes affecting M2 polarization and renal fibrosis to facilitate future targeted therapy; third, before gene lactylation, not only p300 were needed as a lactate transferase, which molecule or molecules acted as lactyl-CoA synthase is worthy for deep exploration.

In summary, our study investigated the role of the gut metabolic product TMAO in CKD. Both in vivo and in vitro experiments revealed that TMAO up-regulates the metabolism of renal intrinsic cells, leading to increased production of lactic acid from pyruvate. This localized increase in lactic acid concentration promotes macrophage M2 polarization and subsequent renal fibrosis. Further exploration revealed that p300 facilitates the lactylation of histone H4K12 on several metabolism-related genes, thereby regulating macrophage activation pathways. This process likely involves a feedback loop on lactic acid production, which requires further validation.

However, our study has some limitations and areas for further investigation. Given that TMAO is known to regulate cholesterol metabolism and atherosclerosis [56], lipid metabolism should be examined in future studies. Moreover, a comprehensive investigation into overall energy metabolism is warranted. Additionally, the specific effects of H4K12la on individual OXPHOS and glycolysis gene expression have not been confirmed and should be addressed in subsequent research. Furthermore, the precise mechanism by which p300 facilitates the localization of lactic acid on gene histones, as well as the specific gene histone lysine sites targeted, remain unclear.

Other histone lysines, such asH1K89la, may also play a role in M2 polarization and merit further investigation.

## Supplementary information

Supplementary figure legends

Supplementary Figure 1

Supplementary Figure 2

Supplementary Figure 3

Supplementary Figure 4

aj-checklist

## Data Availability

The authors declare that all data supporting the conclusions of this study are present in the paper and/or supplementary materials. Additional data supporting the present study are available from the corresponding author upon reasonable request.
