# Supplementary material for: Intestinal metabolite TMAO promotes CKD progression by stimulating macrophage M2 polarization through histone H4 lysine 12 lactylation
Source: Cell Death Differ. 2025 Aug 19;33(2):314–26. doi: 10.1038/s41418-025-01554-z (PMC12881611; doi:10.1038/s41418-025-01554-z)
Supplement: Supplementary file 1 — Supplementary figure legends [file 41418_2025_1554_MOESM1_ESM.docx]

Supplementary Figure legend:

Supplementary figure 1. The mice's body weight and the metabolites in their feces. A, Body weight of different groups of mice. B, Weight of left kidney in different groups of mice. C, The level of TMA, TMAO, Betaine, Creatinine, Carnitine, Choline in faeces of different groups of mice.

Supplementary figure 2. Flow cytometry plots and quantification of MHCII+ macrophage(M1) and CD206+ macrophage(M2) in different groups of mice.

Supplementary figure 3. TMAO induces increased lactate secretion from HK-2 cells. A, Western blotting and RT-qPCR showing the PDH, LDHA levels in HK-2 and HK-2 treated with 200uM TMAO for 48h. B, Elisa showing the Acetyl-CoA, LD level in HK-2 and HK-2 treated with 200uM TMAO for 48h.

Supplementary figure 4. Western blotting full vision included in this research were shown as Actin-1, Actin-2, Collagen I, Fibronectin, H3, H4k12la-1, H4k12la-2, Histone 3, LDHA, LDHA-2, PDH-marked, PDH-2, A-SMA.
