# Supplementary figures and images for: Intestinal metabolite TMAO promotes CKD progression by stimulating macrophage M2 polarization through histone H4 lysine 12 lactylation
Source: Cell Death Differ. 2025 Aug 19;33(2):314–26. doi: 10.1038/s41418-025-01554-z (PMC12881611; doi:10.1038/s41418-025-01554-z)

A

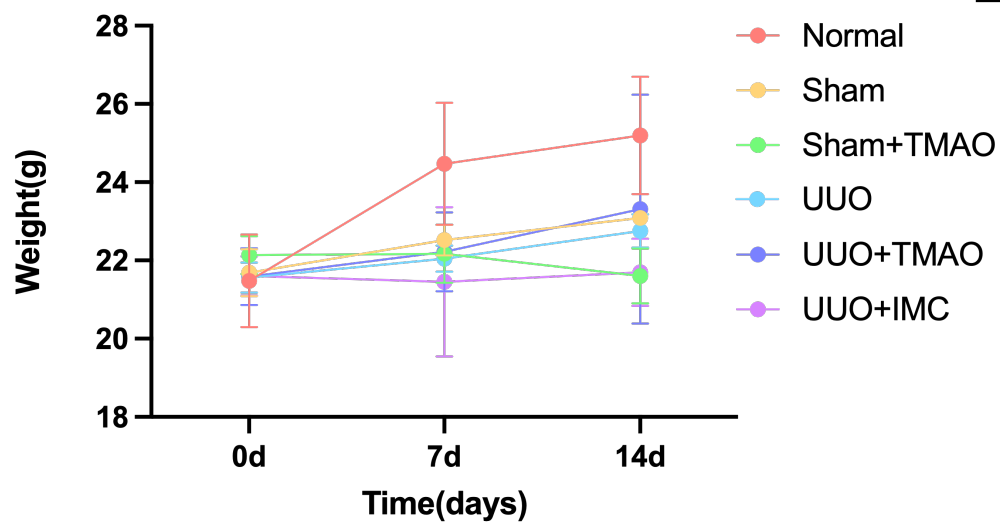

B

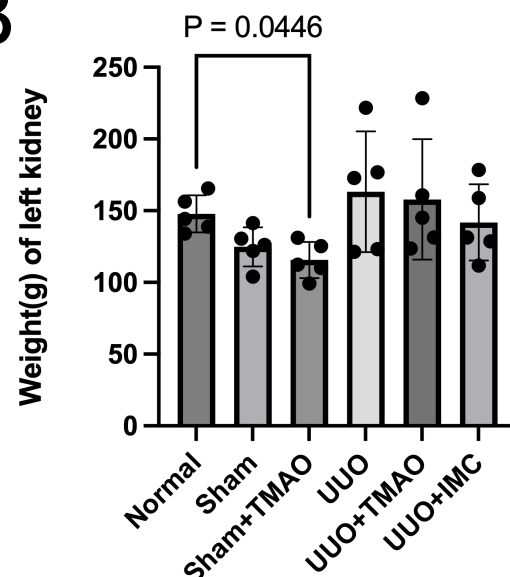

C

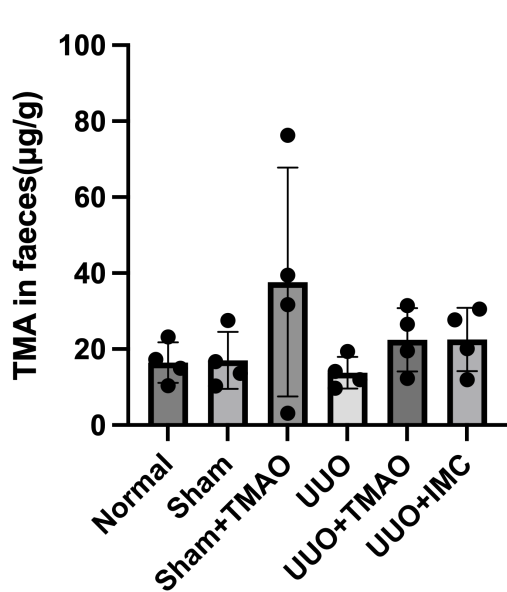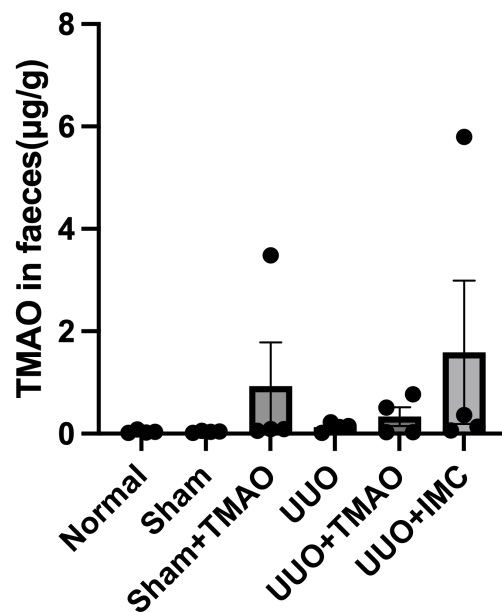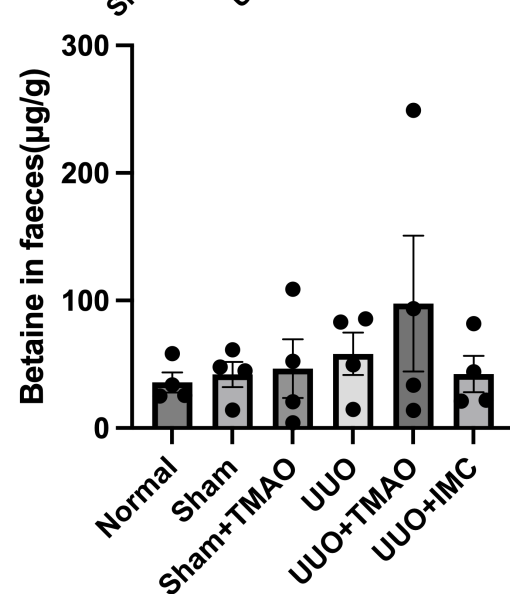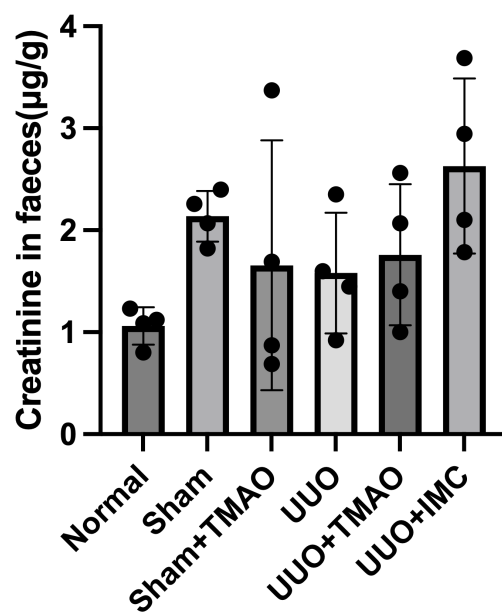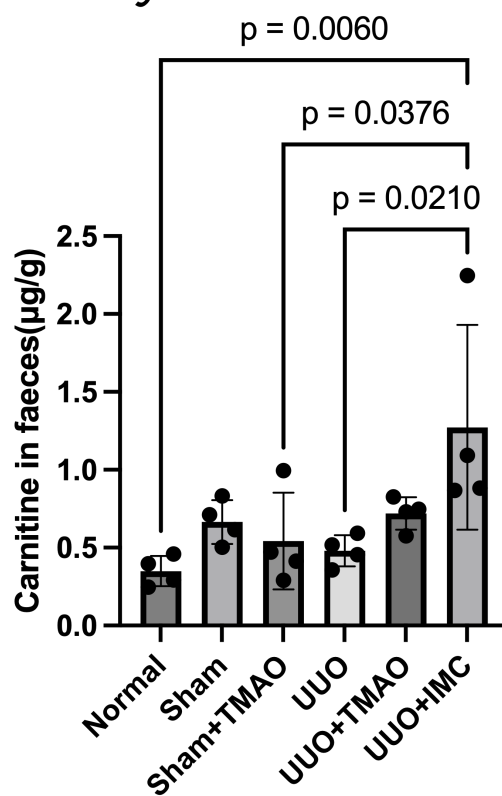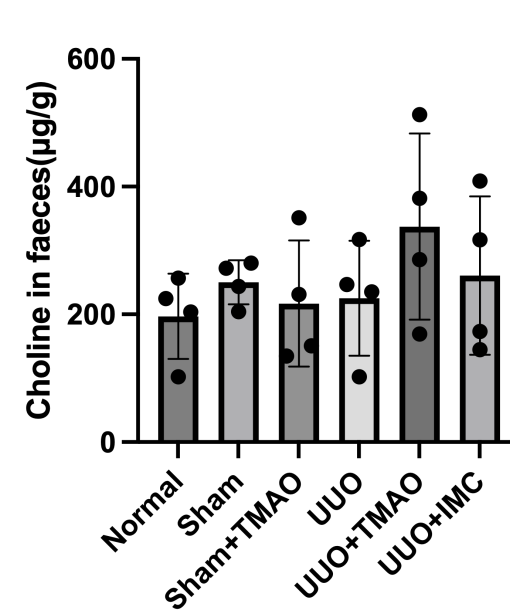

Supplement: Supplementary file 2 — Supplementary Figure 1 [file 41418_2025_1554_MOESM2_ESM.pdf]

A

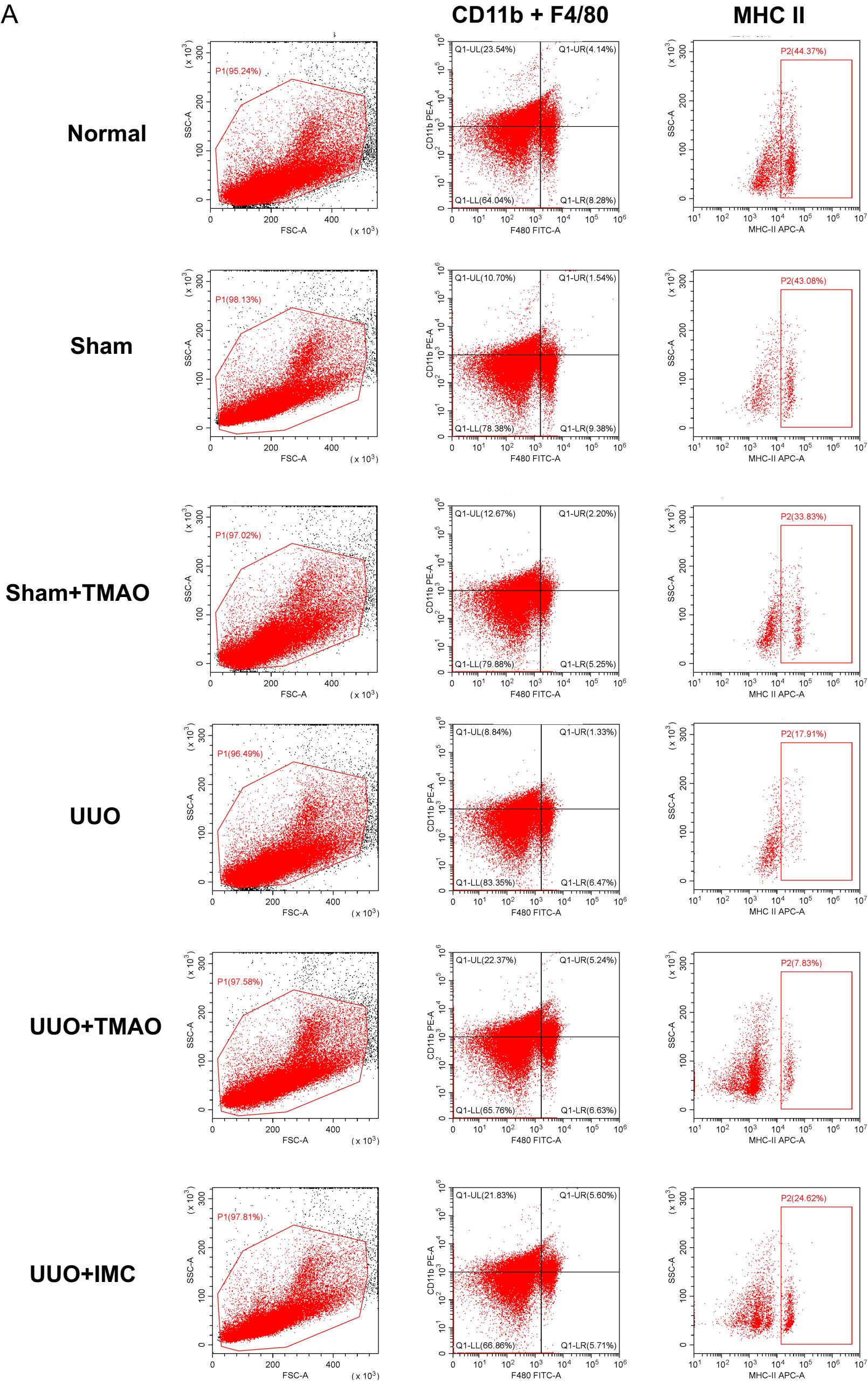

B

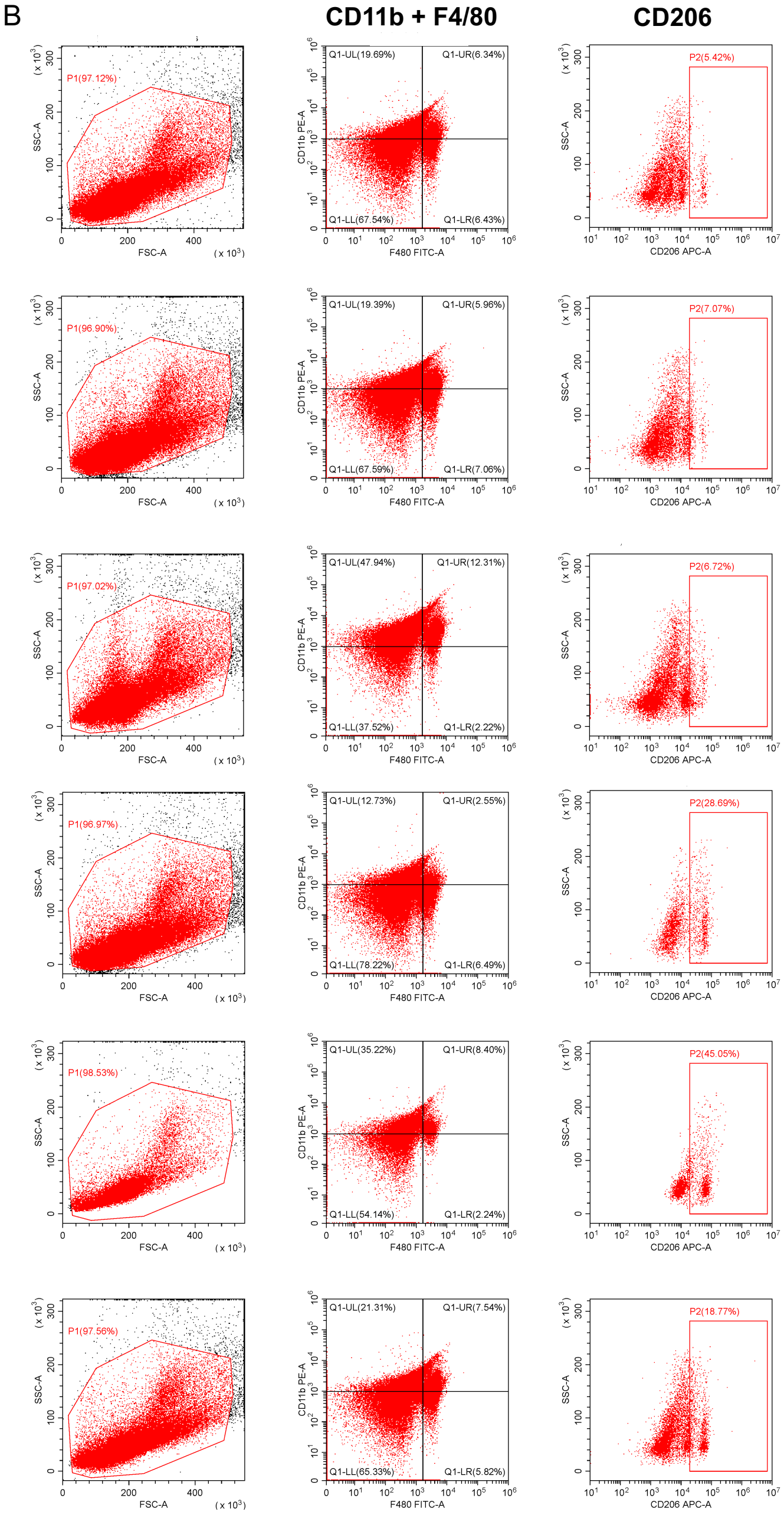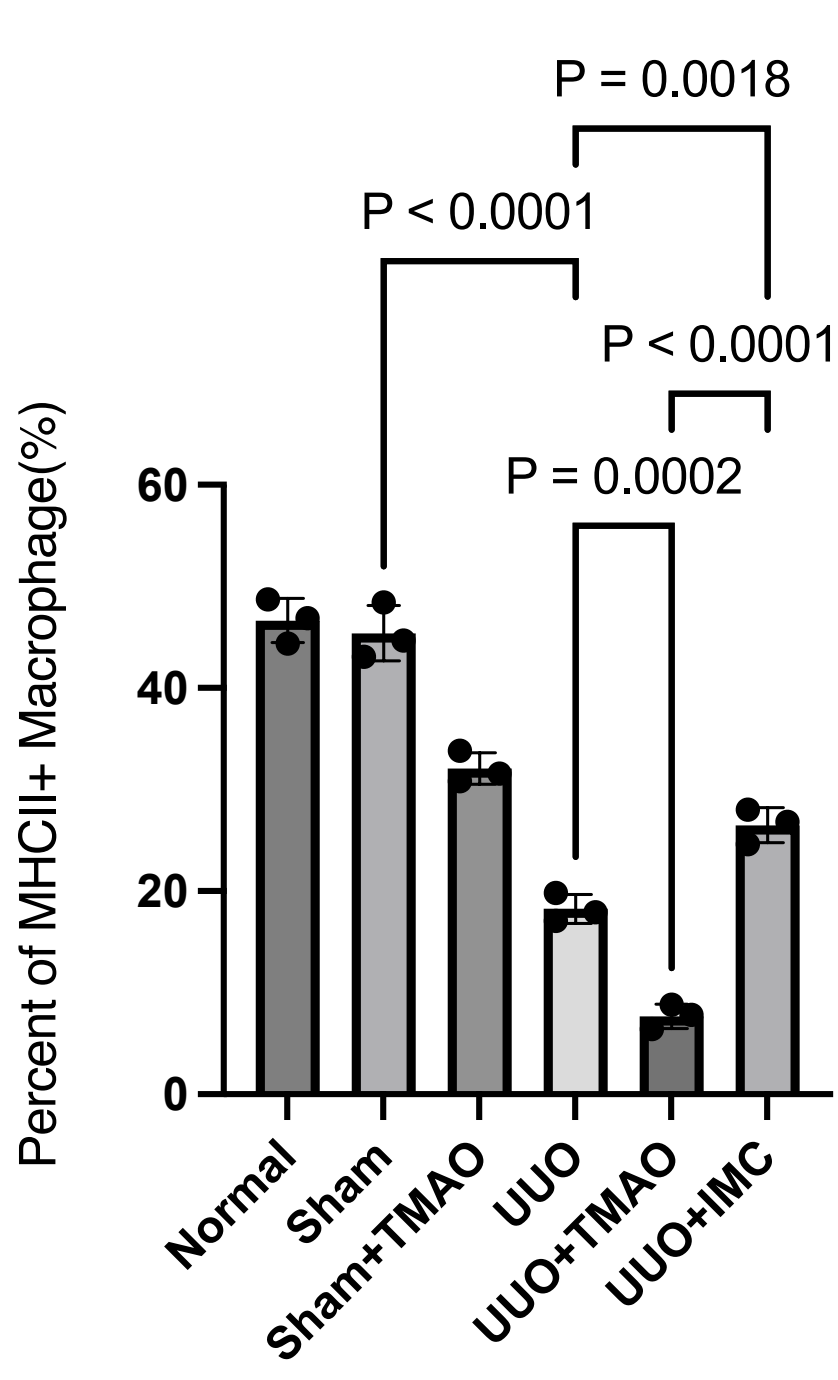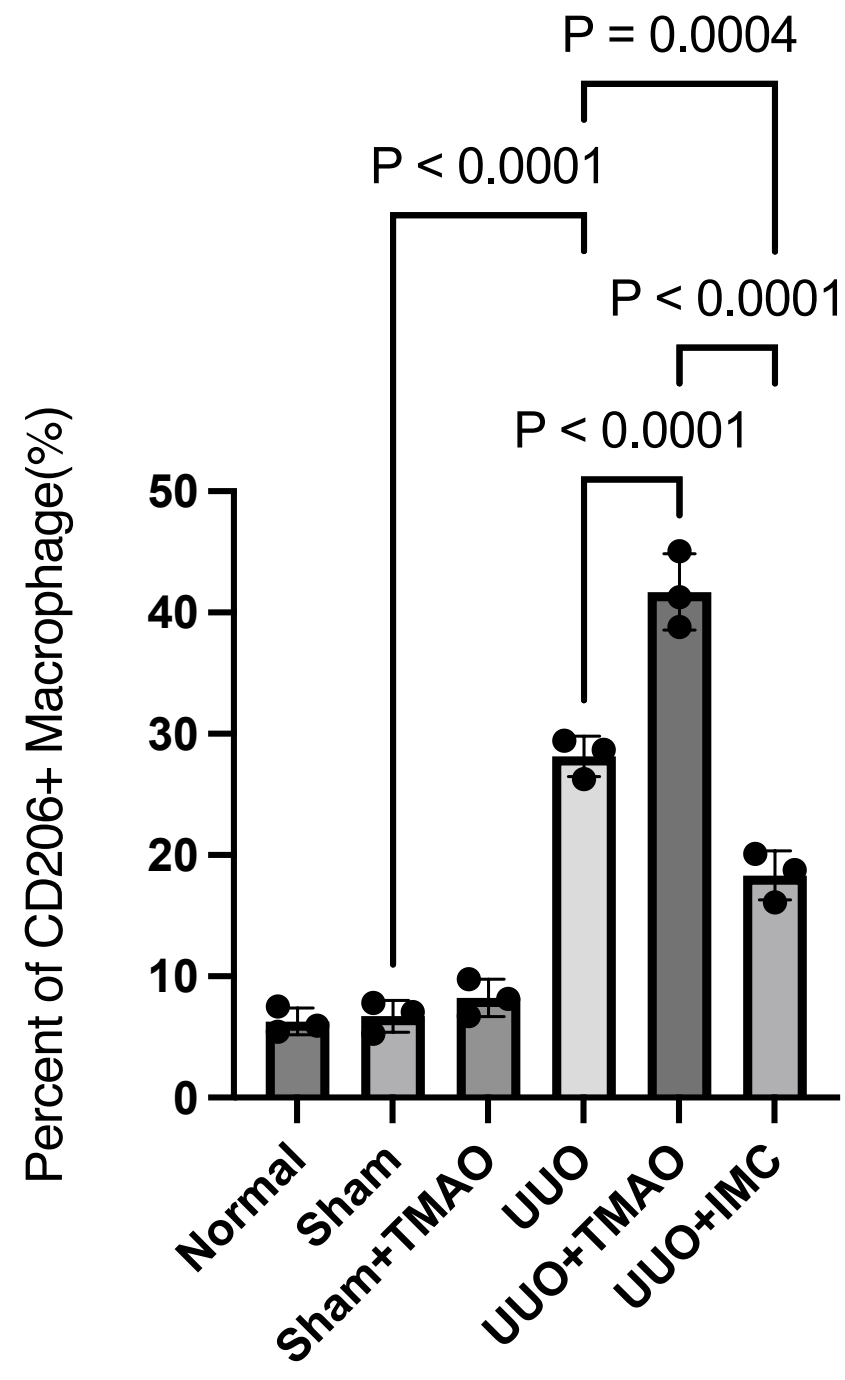

Supplement: Supplementary file 3 — Supplementary Figure 2 [file 41418_2025_1554_MOESM3_ESM.pdf]

A

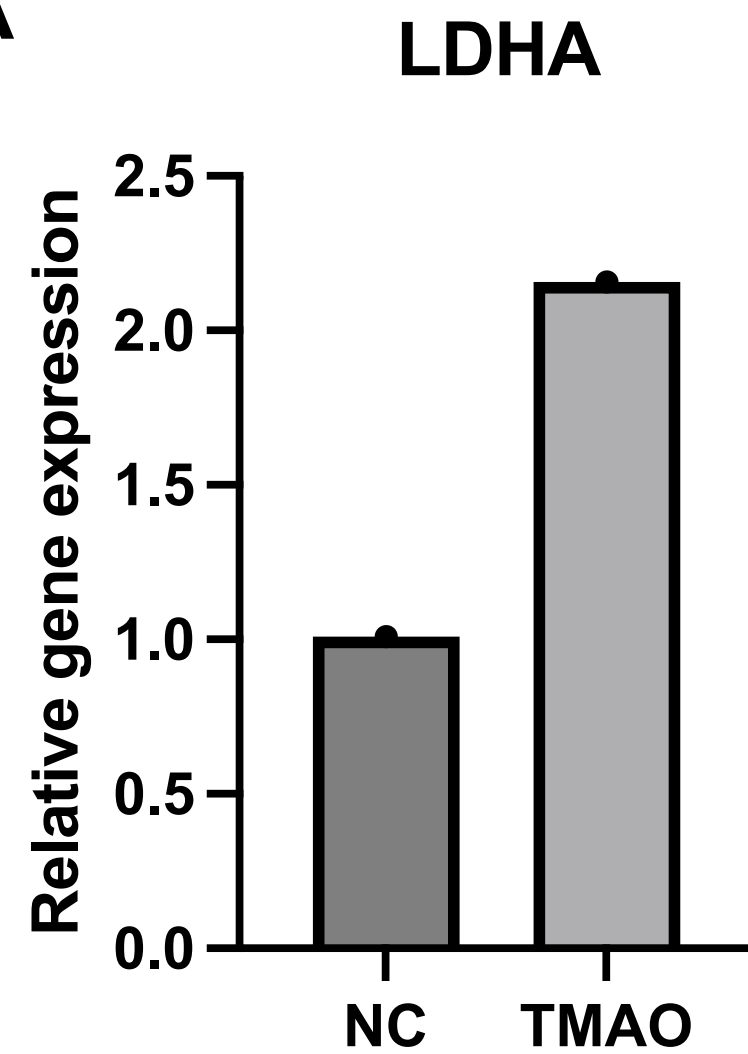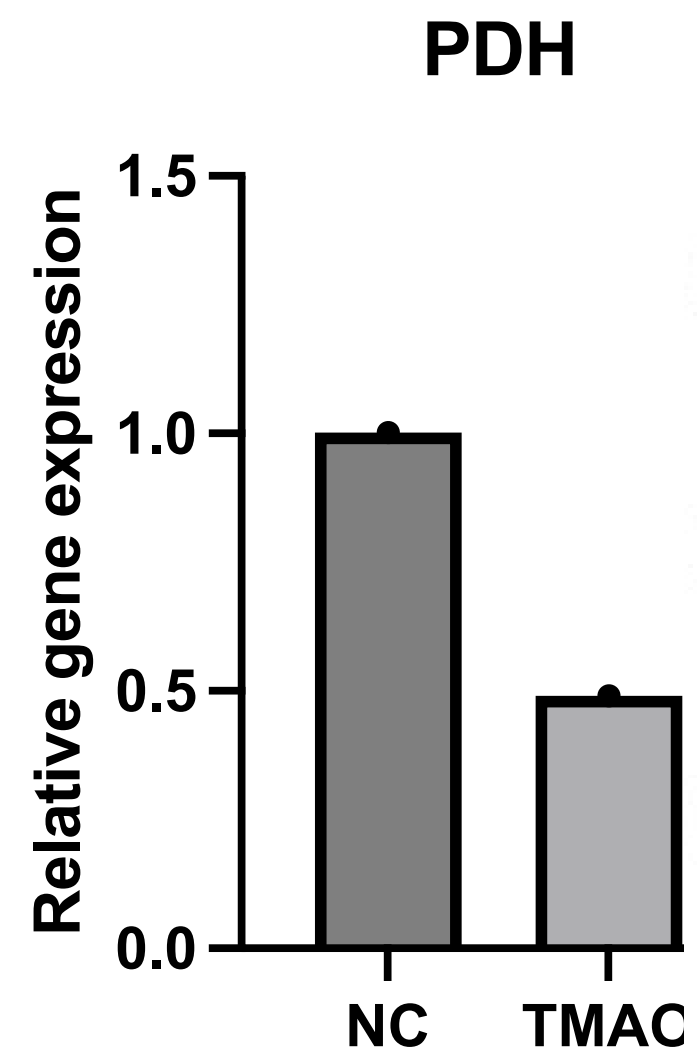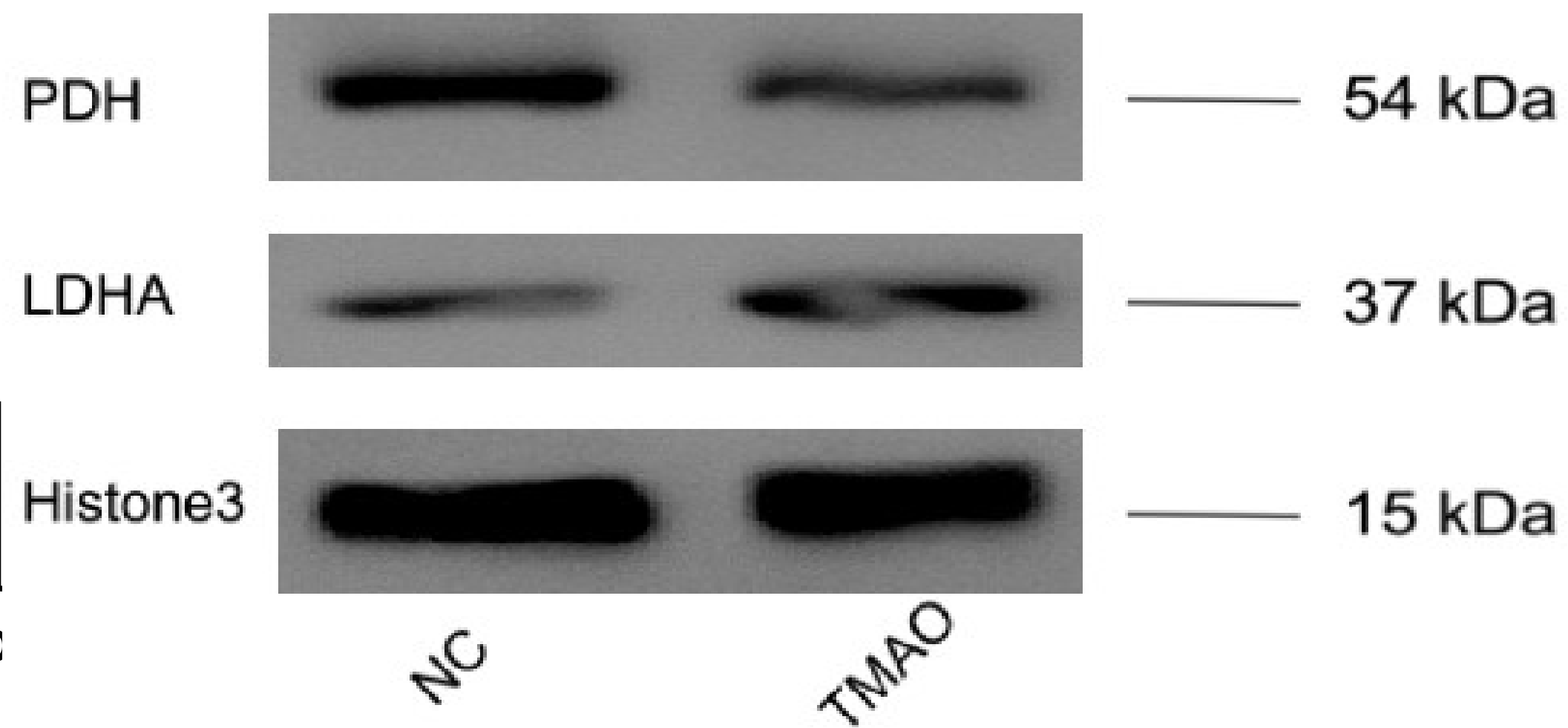

B

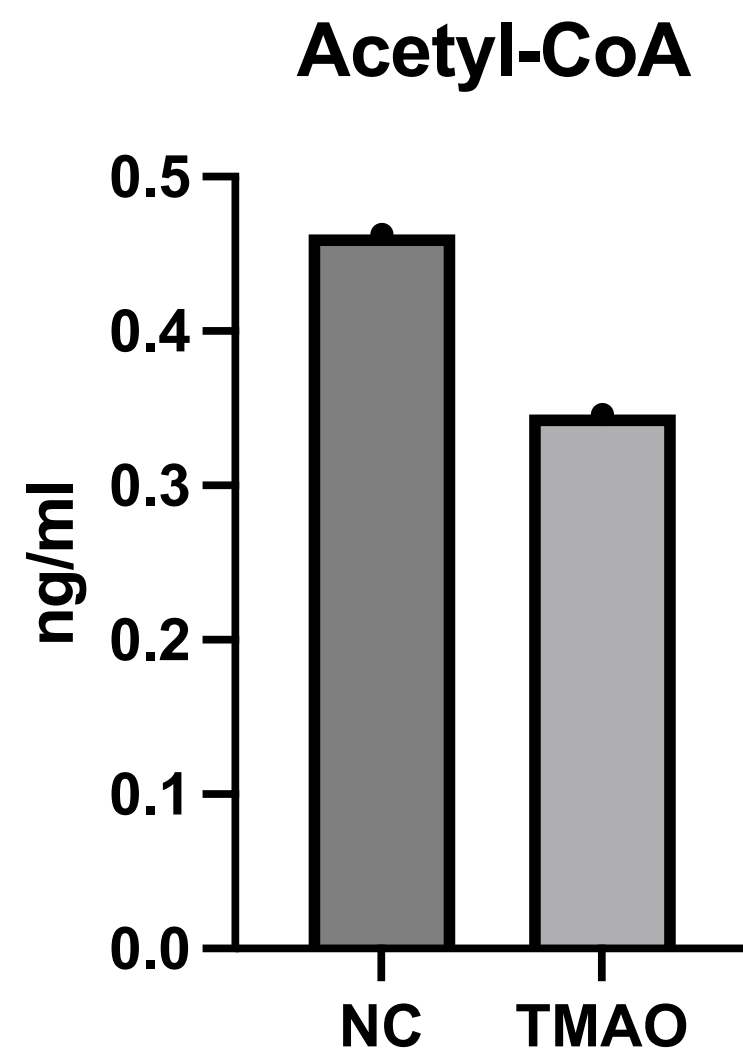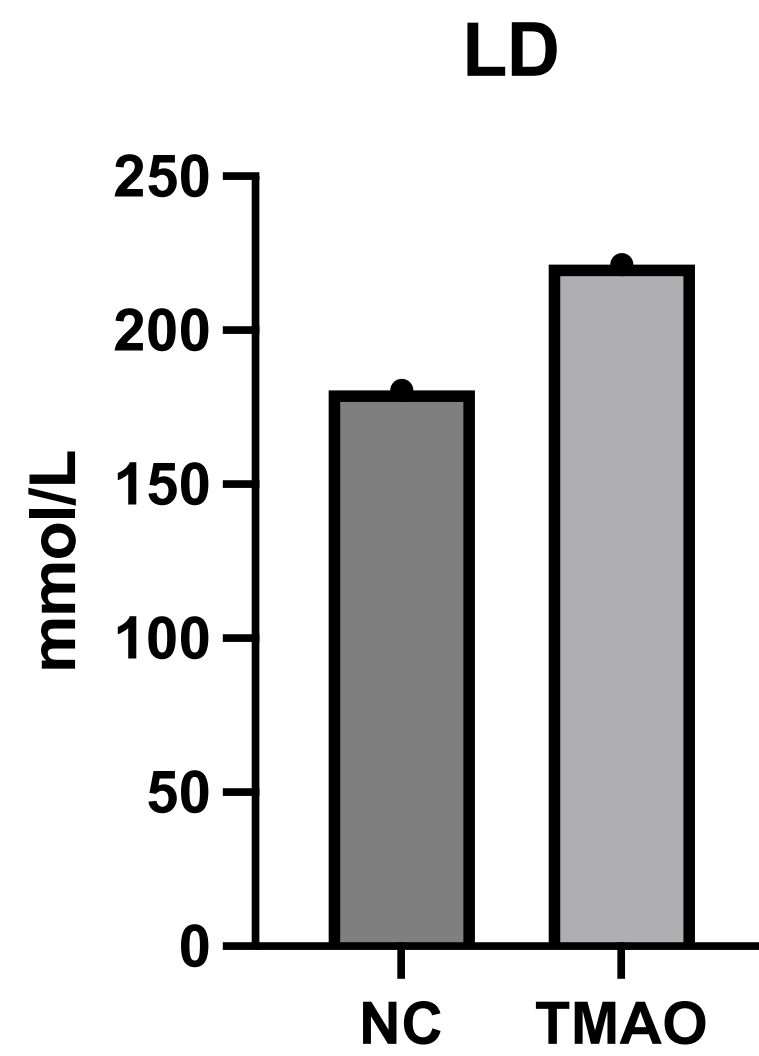

Supplement: Supplementary file 4 — Supplementary Figure 3 [file 41418_2025_1554_MOESM4_ESM.pdf]

Actin-1

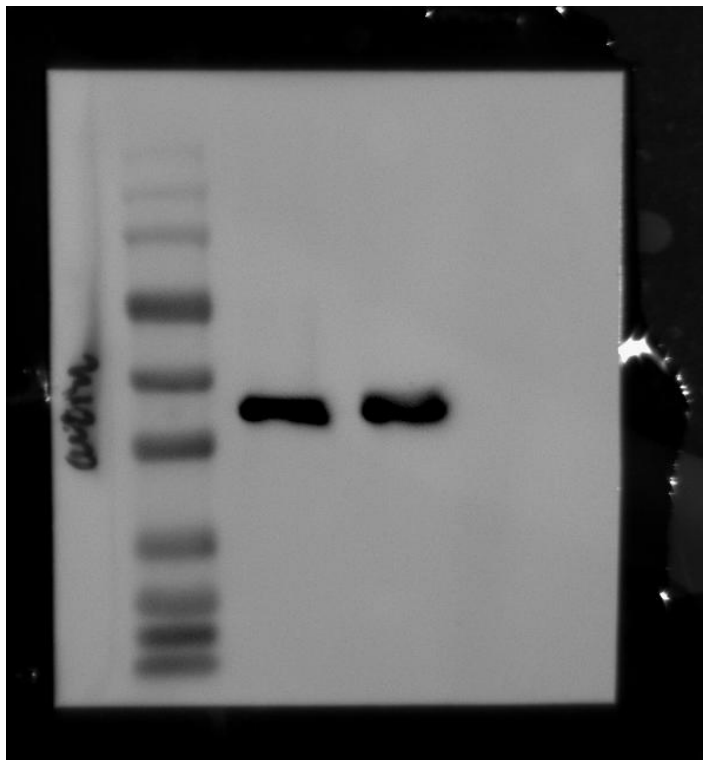

Actin-2

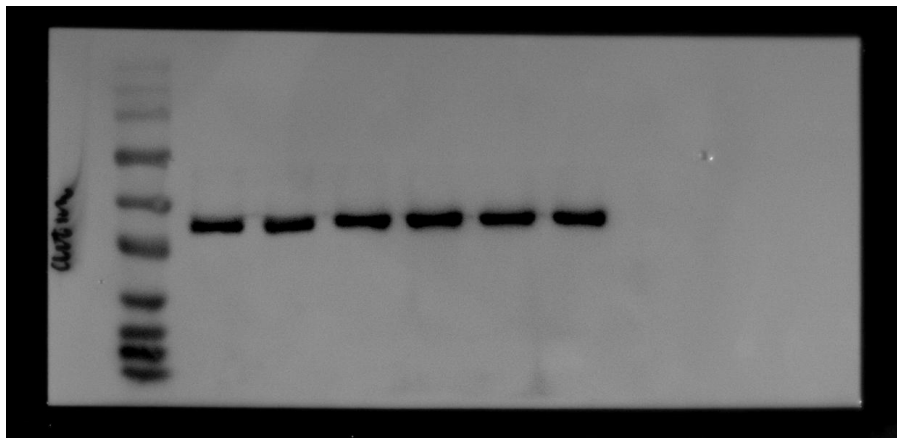

Collagen I

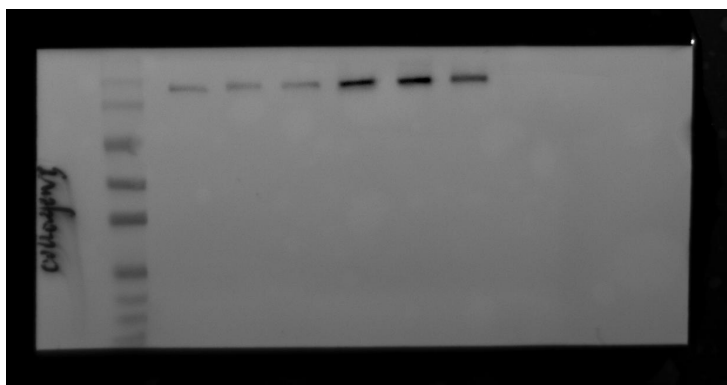

Fibronectin

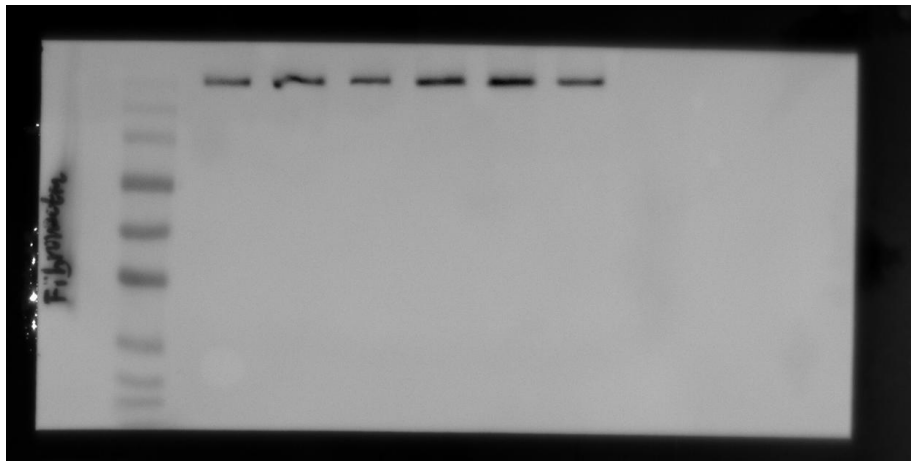

H3

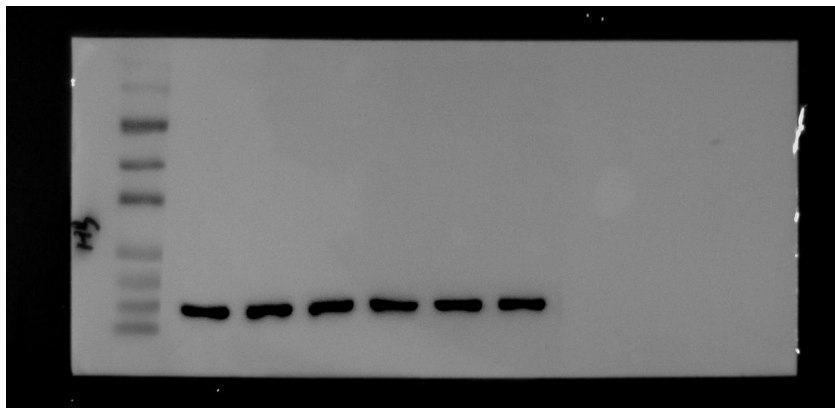

H4K12la-1

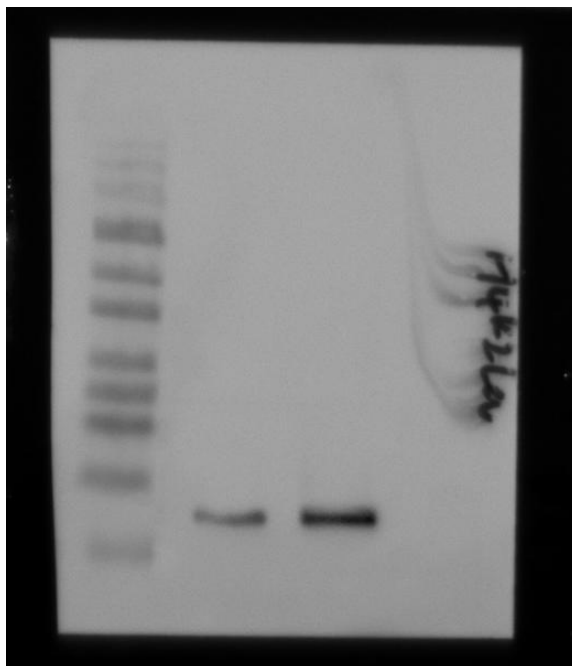

H4K121a-2

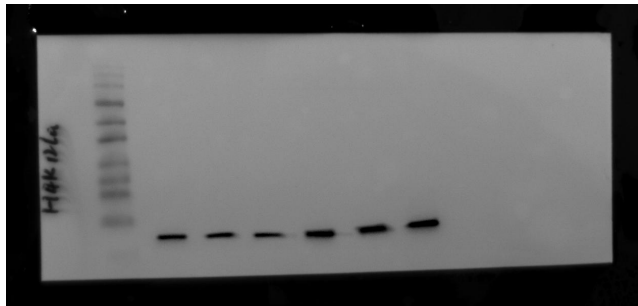

Histone 3

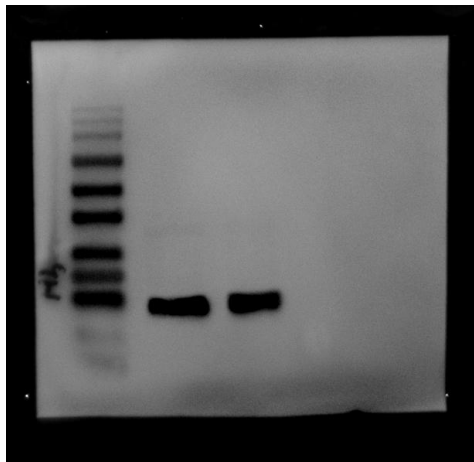

LDHA

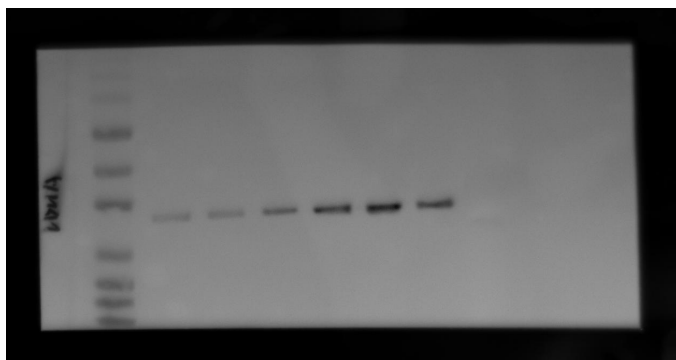

LDHA-2

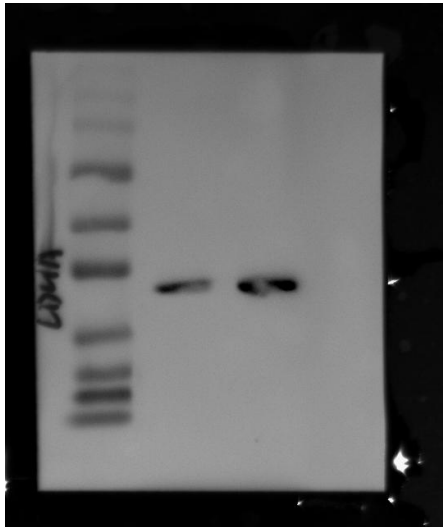

PDH-marked

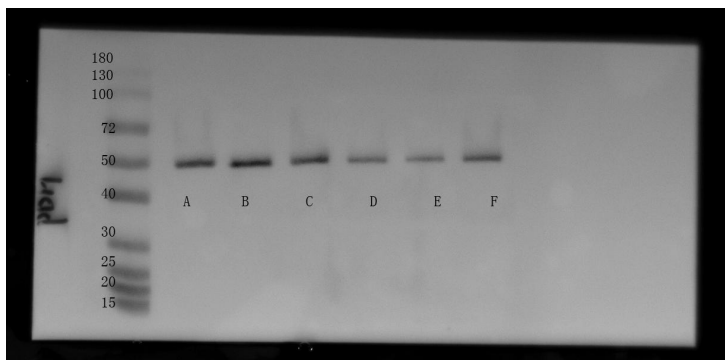

PDH-2

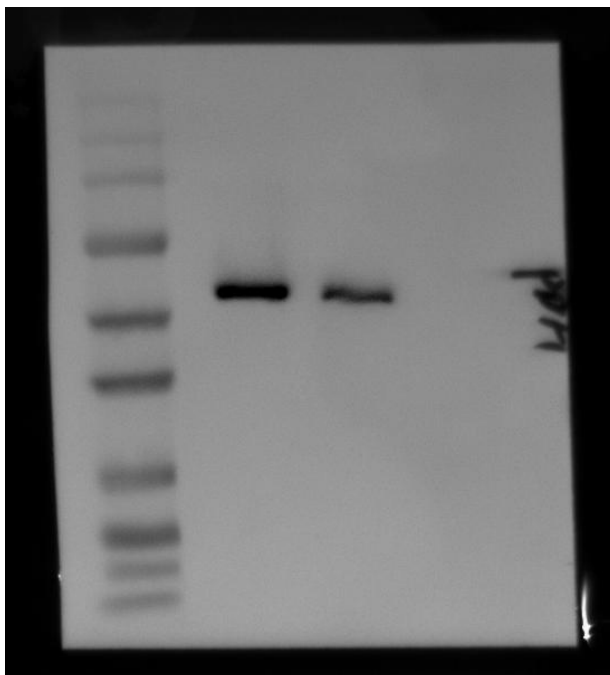

A-SMA

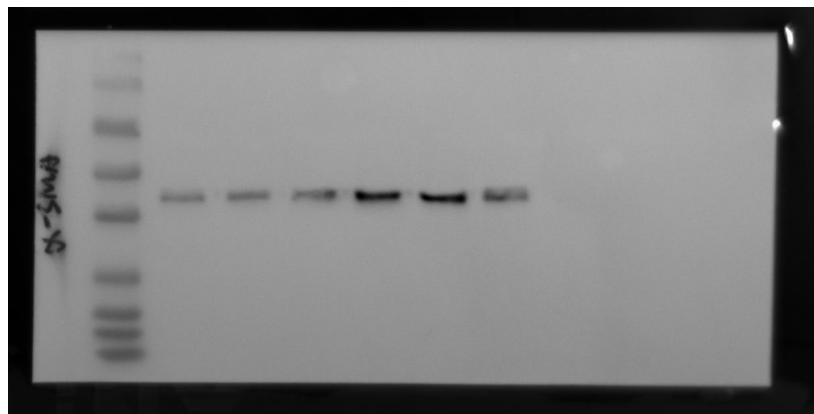

Supplement: Supplementary file 5 — Supplementary Figure 4 [file 41418_2025_1554_MOESM5_ESM.pdf]
